# A Novel Human Tissue-Engineered 3-D Functional Vascularized Cardiac Muscle Construct

**DOI:** 10.3389/fcell.2017.00002

**Published:** 2017-01-30

**Authors:** Mani T. Valarmathi, John W. Fuseler, Jeffrey M. Davis, Robert L. Price

**Affiliations:** ^1^Laboratory of Stem Cell Biology and Tissue Engineering, Department of Comparative Biosciences, College of Veterinary Medicine, University of Illinois at Urbana-ChampaignUrbana, IL, USA; ^2^Department of Pathology, Microbiology and Immunology, School of Medicine, University of South CarolinaColumbia, SC, USA; ^3^Department of Cell Biology and Anatomy, School of Medicine, University of South CarolinaColumbia, SC, USA

**Keywords:** induced pluripotent stem cells, mesenchymal stem cells, embryonic cardiac myocytes, excitation-contraction coupling, dedifferentiation, myocardial regeneration, cardiovascular tissue engineering

## Abstract

Organ tissue engineering, including cardiovascular tissues, has been an area of intense investigation. The major challenge to these approaches has been the inability to vascularize and perfuse the *in vitro* engineered tissue constructs. Attempts to provide oxygen and nutrients to the cells contained in the biomaterial constructs have had varying degrees of success. The aim of this current study is to develop a three-dimensional (3-D) model of vascularized cardiac tissue to examine the concurrent temporal and spatial regulation of cardiomyogenesis in the context of postnatal *de novo* vasculogenesis during stem cell cardiac regeneration. In order to achieve the above aim, we have developed an *in vitro* 3-D functional vascularized cardiac muscle construct using human induced pluripotent stem cell-derived embryonic cardiac myocytes (hiPSC-ECMs) and human mesenchymal stem cells (hMSCs). First, to generate the prevascularized scaffold, human cardiac microvascular endothelial cells (hCMVECs) and hMSCs were co-cultured onto a 3-D collagen cell carrier (CCC) for 7 days under vasculogenic culture conditions. In this milieu, hCMVECs/hMSCs underwent maturation, differentiation, and morphogenesis characteristic of microvessels, and formed extensive plexuses of vascular networks. Next, the hiPSC-ECMs and hMSCs were co-cultured onto this generated prevascularized CCCs for further 7 or 14 days in myogenic culture conditions. Finally, the vascular and cardiac phenotypic inductions were analyzed at the morphological, immunological, biochemical, molecular, and functional levels. Expression and functional analyses of the differentiated cells revealed neo-angiogenesis and neo-cardiomyogenesis. Thus, our unique 3-D co-culture system provided us the apt *in vitro* functional vascularized 3-D cardiac patch that can be utilized for cellular cardiomyoplasty.

## Introduction

Restricted myocardial regeneration after tissue injury and shortage of organs for transplantation are the principal constraints of conventional therapies (Soonpaa and Field, 1998). Organ tissue engineering, including cardiovascular tissues, has been an area of intense investigation. The major challenge to these approaches has been the inability to vascularize and perfuse the *in vitro* engineered tissue constructs (Bursac et al., 1999; Zimmermann et al., 2000; Papadaki et al., 2001). Engineering a tissue of clinically relevant magnitude requires the formation of an extensive and stable microvascular networks within the tissue. Since most *in vitro* engineered tissue constructs do not contain the intricate microvascular structures resembling those of native tissue, the cells contained in scaffolds heavily rely on simple diffusion for oxygenation and nutritional delivery (Zimmermann et al., 2000). Attempts to provide oxygen and nutrients to the cells contained in the biomaterial constructs have had varying degrees of success. Moreover, the interaction of the cells of the host and construct has not been well characterized. Therefore, strategies aiming at the improvement of neovascularization of engineered tissues are of critical importance.

The rate of diffusive transport is crucial for tissue viability, since nutrient delivery must keep up with cellular demand. Fortunately, diffusive transport is very fast over short distances, and impossibly slow over distances greater than a millimeter or so (>100 μm). Thus, there exists a distance limitation of diffusion as transport process (Yamada et al., 1985). As a result, for distances > 100 μm, a faster transport system is clearly needed. The cardiovascular system provides this, at physiological level, the coronary circulation must deliver oxygen at a high rate to match the basal myocardial demand, which is normally 20 times that of resting skeletal muscle. The myocardial capillary density is very high, with the ratio of capillaries to muscle cells approximately 1:1 (3000–5000/mm^2^ section). This structural adaptation of myocardium creates a large endothelial surface area and reduces the maximum diffusion distance to approximately 10 μm (myocytes being 18 to 20 μm), thereby facilitating oxygen and nutrient transfer to the myocytes (Rakusan and Korecky, 1982). This suggests that, on the average, adjacent capillaries are separated by a single muscle cell, consequently, this ensures that myocardial capillary density is greater and diffusion distance becomes correspondingly shorter.

In general, one of the major obstacles for successful cardiovascular tissue engineering is obviously a quantitative one (Ennett and Mooney, 2002; Jain, 2003; Levenberg et al., 2005). The failure of several *in vitro* assembled avascular myocardial tissue constructs to survive implantation into tissue defects is not only due to the inevitable necrosis of the cells in the interior region of the large tissue construct, but also due to an inability to perfuse the tissue construct promptly with vascular sprouts emanating from the host vasculature. Therefore, based on practical experience with “free grafting of tissues” is that cells that are more than 100 to 200 μm from the surface of the graft will experience certain degree of hypoxia or anoxia, and are probably not likely to survive for more than a couple of hours after implantation into the host. In the case of free tissue transplants, the ischemic central region of the graft often becomes revascularized, and the necrotic center of the graft will eventually be repopulated with parenchymal cells that move in with the ingrowing blood vessels (Hölzle et al., 2006; Carlson, 2007). The advent of microvascular surgery resolved many issues that were routinely encountered for tissue grafting, since the modus operandi of connecting the nutrient vessel of the graft to vessels of the host provides instantaneous revitalizing functional blood supply, i.e., the rapid perfusion. Thus, with vascularized tissue grafts, majority of cells of the graft survive, and the tissue regeneration is inevitable due to the avoidance of necrotic events (Hölzle et al., 2006; Carlson, 2007). As a result, this necessitates the formation of appropriate *in vitro* three-dimensional (3-D) plexuses of new blood vessels within the pre-implanted biomaterial constructs through the process of in situ *de novo* vasculogenesis/angiogenesis for organ tissue engineering (Valarmathi et al., 2009).

In this study, we have exploited the developmental biology phenomena, the cell-cell interaction and cell-matrix interaction, and tested the hypothesis that whether functional vascularized cardiac tissue can be generated by the interaction of adult-tissue-derived stem cells., viz., the human induced pluripotent stem cell-derived embryonic cardiac myocytes (hiPSC-ECMs) and the human adipose-derived multipotent mesenchymal stem cells (hMSCs) on a 3-D prevascularized collagen cell carrier (CCC) scaffold.

## Materials and methods

### Priming of CCCs for attachment and cell seeding

CCCs were procured from Viscofan BioEngineering (Weinheim, Germany) (Schmidt et al., 2011). In brief, first, each well of a cell culture treated 24 well plate was preloaded with 250 μl of pre-warmed (37°C) Dulbecco's phosphate-buffered saline (DPBS). Next, a sterile CCC was placed onto the liquid containing wells using a sterile forceps, avoiding entrapment of air bubbles. Each one of those CCCs was submerged with a blunt forceps, and incubated for 5 min at 37°C. The DPBS was removed and CCCs were washed twice, and were dried overnight in an operating laminar flow hood with the lid removed or ajar. After drying, the CCCs were attached firmly to the bottom of the well, and was ready for cell seeding. Prior to cell seeding, the CCCs were equilibrated by incubation with appropriate volume of the desired pre-warmed culture medium for at least 10 min at 37°C. Finally, the medium was removed, and the CCCs were subjected to physical crosslinking using Stratalinker® UV Crosslinker 1800 (Stratagene), prior to seeding cells onto the CCCs.

### hCMVECs culture: plating, maintenance, and subculture

Human cardiac microvascular endothelial cells (hCMVECs) were purchased from Lonza (Walkersville, MD), and subcultured according to manufacturer's recommendations. Briefly, cells were thawed and seeded on a fibronectin precoated T75 flasks (1 μg/cm^2^ or 0.5 μg/mL) and expanded using complete microvascular endothelial cell growth medium, supplemented with 5% fetal bovine serum (FBS), human epidermal growth factor (hEGF), vascular endothelial growth factor (VEGF), R3-insuling-like growth factor-1 (R3-IGF-1), human fibroblast growth factor-beta (hFGF-β), ascorbic acid, hydrocortisone, gentamicin, and amphotericin-B (Clonetics™ EGM™-2MV BulletKit™; Lonza).

### hiPSC-ECMs culture: plating and maintenance

hiPSC-ECMs were procured from Cellular Dynamics International (iCells; CDI, Madison, WI), and were cultured as per the manufacturer's recommendations. Initially, the cells were thawed in specially prepared iCell cardiomyocyte plating medium, and were seeded onto 0.1% (w/v) gelatin-coated six-well tissue-culture plates at a density of 7.5 × 10^5^ to 1.0 × 10^6^ cells/well. Two days after plating, the plating medium was replaced with iCell cardiomyocyte maintenance medium. Next, the culture medium was changed with fresh medium every other day. The cells were cultured for 2 to 7 days after the initial thawing at 37°C with 5% CO_2_ prior to re-plating.

### hMSCs culture: plating, maintenance, and subculture

hMSCs were obtained from ScienCell Research Laboratories (Carlsbad, CA), and were expanded and maintained as per the manufacturer's instruction. After three passages, the attached hMSCs were trypsinized and subjected to further purification and characterization.

### Immunophenotyping of hMSCs by multi-color flow cytometry and immunofluorescence microscopy

Qualitative analysis for a number of cell surface markers (BD Stemflow™ human MSC analysis kit) was performed on cells that were grown in the Lab-tek™ chamber slide system™ (Nunc) using a Olympus BX53 fluorescence microscope system (Olympus Corporation), and quantitative analysis of the same set of surface markers was performed by multi-color flow cytometry using a BD™ LSR II Flow Cytometer (Beckman, Dickinson and Company) as described by manufacture's instruction (Reyes et al., 2002; Dominici et al., 2006; Valarmathi et al., 2009).

Briefly, hMSCs were harvested by trypsinization, centrifuged at 200 g for 5 min. The pelleted cells were washed two times with DPBS solution, pH 7.4, and were re-suspended in 1.5% BSA (bovine serum albumin in DPBS, pH 7.4), at a concentration of 1 × 10^7^ cells. The re-suspended single cells were incubated at 4°C for 25 min with appropriate dilutions of fluorochrome-conjugated mouse anti-human (hMSC positive cocktail: FITC CD90/PerCP-Cy™5.5 CD105/APC CD73) (Table 1) monoclonal antibodies for direct immunostaining. Likewise, the cells were incubated with fluorochrome-conjugated mouse anti-human (hMSC negative cocktail: PE CD11B/PE CD19/PE CD34/PE CD45/PE HLA-DR) (Table 1). Appropriate fluorochrome-labeled mouse IgGs antibodies served as the isotype controls for hMSC positive and negative cocktails. The stained cells were washed two times with DPBS solution, and the cells were fixed in ice-cold 0.5% paraformaldehyde (PFA) and stored in the dark at 4°C until acquired in flow cytometry. Finally, the acquired raw data were analyzed using BD FACSDiva, version 8 software.

**Table 1:** **Primary antibodies used in this study**.

**Primary antibodies**	**Dilutions**	**Source**	**Cell target**
**hMSCs CHARACTERIZATION MARKERS**			
CD11B	1:50	Beckman Dickinson	Leukocytes
CD19	1:50	Beckman Dickinson	Leukocytes
CD34	1:50	Beckman Dickinson	Hematopoietic
CD45	1:50	Beckman Dickinson	Leukocytes
CD73	1:50	Beckman Dickinson	MSCs
CD90	1:50	Beckman Dickinson	MSCs
CD105	1:50	Beckman Dickinson	MSCs
HLA-DR	1:50	Beckman Dickinson	Leukocytes
**ENDOTHELIAL CELL DIFFERENTIATION MARKERS**			
PECAM1	1:100	Santa Cruz Biotechnology	Endothelial
VWF	1:100	Santa Cruz Biotechnology	Endothelial
VE-CADHERIN	1:100	Santa Cruz Biotechnology	Endothelial
LECTIN	1:50	Vector Laboratories	Endothelial
LAMININ	1:200	Abcam	Endothelial
**SMOOTH MUSCLE CELL DIFFERENTIATION MARKERS**			
α-SMA	1:100	Sigma-Aldrich	Smooth Muscle
**CARDIAC MYOCYTE DIFFERENTIATION MARKERS**			
CARDIAC MYOSIN HEAVY CHAIN (α/β MHC)	1:200	Abcam	Cardiomyocyte
CARDIAC TROPONIN I (cTnI)	1:200	Santa Cruz Biotechnology	Cardiomyocyte
N-CADHERIN	1:200	Santa Cruz Biotechnology	Cardiomyocyte
CONNEXIN 45 (Cx45)	1:200	Santa Cruz Biotechnology	Cardiomyocyte
CONNEXIN 43 (Cx43)	1:200	Santa Cruz Biotechnology	Cardiomyocyte
DESMIN	1:200	Abcam	Cardiomyocyte
GATA4	1:200	Santa Cruz Biotechnology	Cardiomyocyte
BNP	1:200	Santa Cruz Biotechnology	Cardiomyocyte

### Enrichment of hMSCs by magnetic-activated cell sorting (MACS)

Additional purification and enrichment of the cultured hMSCs were performed as per our previously published method (Valarmathi et al., 2010), using an autoMACS™ Pro Separator (Miltenyi Biotech). The resulting enriched CD45^−^/CD34^−^/CD90^+^ fractions were subcultured and expanded further (Valarmathi et al., 2010).

### Labeling of hMSCs with red or green fluorescent protein (RFP or GFP) for cell lineage tracing

#### Lentiviral vectors construction and lentivirus assembly

Lentiviral vectors construction and lentivirus production were carried out as per our previously published protocol (Valarmathi et al., 2011), using pWPI-RFP or pWPT-GFP, together with pCMVR8.74 (packaging plasmid) and pMD2.G (envelope plasmid). And finally, the viral titre was ascertained by standard HeLa titre procedure using either RFP or GFP as a marker.

#### hMSCs lentiviral transduction

Lentiviral transduction of hMSCs (0.2 × 10^6^ cells/well) using the desired number of viral particles (MOI = 5) were performed precisely using the previously published protocol (Valarmathi et al., 2011), and the transduction efficiency was estimated using single color FACS, and was greater than 95%.

### Generation of vascularized CCCs

#### Seeding of hCMVECs onto CCCs

Next, subcultured and expanded hCMVECs were plated onto the prepared and fibronectin (1 μg/cm^2^ or 0.5 μg/mL) precoated CCCs at a density of 0.5 × 10^6^ cells/30 mm CCC and cultured in complete microvascular endothelial cell growth medium (Clonetics™ EGM™-2MV BulletKit™; Lonza) for either 7 or 14 days.

#### Seeding of hCMVECs/hMSCs onto CCCs

In addition, hCMVECs were co-cultured with hMSCs (0.5 × 10^6^ of hCMVECs and 0.3 × 10^6^ of hMSCs/30 mm CCC) and cultured in complete microvascular endothelial cell growth medium (Clonetics™ EGM™-2MV BulletKit™; Lonza) for either 7 or 14 days.

The cultures (hCMVECs or hCMVECs/hMSCs) were terminated at these regular intervals (day 7 or day 14), and the collected samples were subjected to RT-qPCR, immunofluorescence, ultrastructural, and biochemical analyses.

### Functional characterization of vascularized CCCs by Dil-Ac-LDL uptake assay

Functional characterization of endothelial cells (i.e., CCCs seeded with either hCMVECs or hCMVECs/hMSCs and cultured under vasculogenic differentiation conditions for 7 or 14 days) were carried out using Dil-conjugated acetylated low-density lipoproteins (Dil-Ac-LDL staining kit, Biomedical Technologies, Inc.), as described previously (Voyta et al., 1984). Subsequently, immunostaining of the CCCs with VE-cadherin antibody (1:200 in staining buffer) was also performed to demarcate the endothelial cells (as described in immunofluorescence staining section below). DAPI (100 ng/ml) was used to counterstain the nuclei. The images of Dil- and VE-cadherin-labeled endothelial cells were captured using an Olympus BX53 fluorescence microscope system.

### Scanning electron microscopic (SEM) analysis of vascularized CCCs

To depict the nature and structural organization of the vascular component, the Day 14 CCC samples (i.e., the CCCs that were cultured in vasculogenic culture conditions, viz., hCMVECs/hMSCs) were processed for SEM by means of the O-GTA-O-GTA-O method (Hanaichi et al., 1986).

### Generation of vascularized cardiac patch

#### Seeding hiPSC-ECMs onto CCCs

hiPSC-ECMs were quantified using a hemocytometer and plated at a density of 1 × 10^6^ cells/well of a 24 well-bottom culture dish that was pre-attached with individual CCCs, incubated in a humidified atmosphere of 5% CO_2_ at 37°C for 48 h, and observed under inverted phase contrast microscope (Olympus IX 73) for spontaneous beating and rhythmic contractions. These hiPSC-ECMs were cultured in complete myocyte medium (DMEM with 8% horse serum, HS, and 5% newborn calf serum, NCS) for further 7 or 14 days.

#### Seeding hiPSC-ECMs and hMSCs onto CCCs

The purified and enriched population of hMSCs (CD90^+^) (GFP unlabeled or GFP labeled) were plated onto the surface of the previously produced hiPSC-ECMs CCC's wells (after 48 h) at a density of 0.4 × 10^5^ cells/well, and were cultured in complete myogenic medium for 7 or 14 days.

The cultures (hiPSC-ECMs only or hiPSC-ECMs/hMSCs-GFP unlabeled or hiPSC-ECMs/hMSCs-GFP labeled) were terminated at these regular intervals (day 7 or day 14). And the collected samples were subjected to RT-qPCR, immunofluorescence, ultrastructural, calcium transit, as well as pharmacological analyses.

#### Seeding hiPSC-ECMs and hMSCs onto prevascularized CCCs

The hiPSC-ECMs (1 × 10^6^ cells/well) and hMSCs (0.4 × 10^5^ cells/well) were simultaneously added on top of prevascularized CCCs that were cultured in vasculogenic medium for 7 days, i.e., CCCs that were created by the combination co-culture of hCMVECs/hMSCs only. And cultured further in complete myogenic medium for 7 or 14 days.

Finally, the vascularized cardiac CCCs (cardiac patch), now containing all three categories of cells, viz., hCMVECs, hiPSC-ECMs, and hMSCs were terminated at these regular intervals (day 7 or day 14), and the collected samples were subjected to immunofluorescence staining and confocal microscopic analysis to validate the simultaneous presence of both vascular and muscular components in these CCC cardiac patches.

### hiPSC-ECMs and hMSCs CCCs contractility and calcium flux assays

#### Loading of cells with calcium orange or fluo-4 calcium-indicators

Loading of co-differentiating cells (CCC cultures—hiPSC-ECMs only or hMSCs-GFP only or hiPSC-ECMs/hMSCs-GFP labeled) with Fluo-4 or Calcium Orange calcium-indicator was carried out as described previously (Valarmathi et al., 2011). The live cell imaging using the spinning disk confocal microscopy was employed to record the changes in the intracellular Ca^2+^ flux of these labeled cells.

#### Live cell imaging using spinning disk confocal microscope

Ultimately, changes in intracellular calcium flux (all types of cells in the CCCs) were examined as elaborated previously (Valarmathi et al., 2011), and by using the AQM Advance-6 software.

### Transmission electron microscopic (TEM) analysis of CCCs

To elucidate the ultrastructural characteristics of co-differentiating cells, day 14 CCC samples (i.e., CCCs that were seeded with cells, hMSCs only or hiPSC-ECMs only or hMSCs/hiPSC-ECMs or hCMVECs/hMSCs, and cultured using appropriate media) were processed for TEM analysis as described elsewhere (Valarmathi et al., 2010).

### Reverse transcription-quantitative real-time polymerase chain reaction (RT-qPCR)

Total cellular RNA isolation from three independent CCC cultures of various combinations (vasculogenic: hCMVECs culture, hCMVECs/hMSCs co-culture; cardiomyogenic: hiPSC-ECMs culture, hiPSC-ECMs/hMSCs co-culture) that were maintained either in vasculogenic medium or myogenic medium were done using TRIzol® Plus RNA purification kit (Invitrogen) as per manufacturer's instructions.

The RNA integrity (RIN) of the extracted samples was analyzed on the Agilent 2100 Bioanalyzer system using the Agilent RNA 6000 nano kit (Agilent Technologies, Inc.) following the manufacturer's recommendations. The reverse transcriptase (RT) reaction was executed using 250 ng of total RNA in a final reaction volume of 20 μl using an iScript™ Reverse Transcription Supermix for RT-qPCR kit (Bio-Rad Laboratories, Inc.) according to the manufacturer's protocols.

The cardiomyogenic gene-specific primers for MYH6 (myosin heavy chain 6), MYH7 (myosin heavy chain 7), ACTC1 (actin, alpha, cardiac muscle 1), TNNI3 (troponin I3, cardiac type), GATA4 (GATA binding protein 4), NPPA (natriuretic peptide A), NPPB (natriuretic peptide B), and GJA1 (gap junction protein, alpha 1); and the vasculogenic gene-associated primers for PECAM1 (platelet and endothelial cell adhesion molecule 1), KDR (kinase insert domain receptor, a type III receptor tyrosine kinase), TIE1 (tyrosine kinase with immunoglobulin-like and EGF-like domains 1), TEK (TEK tyrosine kinase, endothelial), and VWF (von Willebrand factor); as well as the endogenous normalizer reference genes, GAPDH (glyceraldehyde-3-phosphate dehydrogenase), β-ACTIN (cytoplasmic beta-actin), G6PD (glucose-6-phosphate dehydrogenase, and RPLP0 (ribosomal protein lateral stalk subunit P0) were designed using web based software Primer3 (Rosen and Skaletsky, 2000), synthesized commercially (Integrated DNA Technologies, Inc.), and evaluated for an uniform annealing temperature of 58°C, for all the primer pairs, as shown in Table 2.

**Table 2:** **RT-qPCR primer sequences used in this study**.

**Genes**	**Forward primer**	**Reverse primer**	**Product length (bp)**	**Annealing temperature (°C)**	**GenBank accession No**.
**CARDIOMYOGENIC SPECIFIC GENES**					
MYH6	5′–AAGACTGTGAACACCAAGCG–3′	5′–TGTTCGCATTGGCATTGTCC–3′	91	58	NM_002471.3
MYH7	5′-ACATGCTGCTGATCACCAAC-3′	5′-AAGCGTTATCAGTGGCCATG-3′	111	58	NM_000257.2
ACTC1	5′–ATGTCGCCCTGGATTTTGAG–3′	5′–AGCGCTCATTGCCAATAGTG–3′	111	58	NM_005159.4
TNNI3	5′-CGACATAGAGGCAAAAGTCACC-3′	5′-GCTTAAACTTGCCTCGAAGGTC-3′	86	58	NM_000363.4
GATA4	5′-TGTCAACTGTGGGGCTATGTC-3′	5′-TGCCGTTCATCTTGTGGTAGAG-3′	98	58	NM_002052.3
NPPA	5′-GTGAGCTTCCTCCTTTTACTGG-3′	5′-AATCCATCAGGTCTGCGTTG-3′	94	58	NM_006172.3
NPPB	5′-ACCGCAAAATGGTCCTCTACAC-3′	5′-TCCATCTTCCTCCCAAAGCAG-3′	85	58	NM_002521.2
GJA1	5′-TGTGGACATGCACTTGAAGC-3′	5′-TGATGTAGGTTCGCAGCAAC-3′	104	58	NM_000165.3
**VASCULOGENIC SPECIFIC GENES**					
PECAM1	5′-TGGCAACTACACGTGCAAAG-3′	5′-AAGATTCCAGTTCGGGCTTG-3′	101	58	NM_000442.4
KDR	5′-TGGCCAAGTGATTGAAGCAG-3′	5′-ATGCTCACTGTGTGTTGCTC-3′	103	58	NM_002253.2
TIE1	5′-ACGCAGCCATCAAAATGCTG-3′	5′-TGCCCCAATTTGCACAGAAC-3′	91	58	NM_005424.4
TEK	5′-AGAATGCATTTGCCCTCCTG-3′	5′-AAGTTCTGCCAAACGTGTGC-3′	77	58	NM_000459.3
VWF	5′-AGAAAGCCCATTTGCTGAGC-3′	5′-AAGTATCGCACAGCAAAGCC-3′	94	58	NM_000552.3
**REFERENCE GENES**					
GAPDH	5′-AATTCCATGGCACCGTCAAG-3′	5′-ATCGCCCCACTTGATTTTGG-3′	104	58	NM_002046.4
β-ACTIN	5′-TCGTGCGTGACATTAAGGAG-3′	5′-TTGCCAATGGTGATGACCTG-3′	133	58	M10277.1
G6PD	5′-TCATCATCATGGGTGCATCG-3′	5′-AAGGTGTTTTCGGGCAGAAG-3′	97	58	NM_000402.4
RPLP0	5′–AGAACACCATGATGCGCAAG–3′	5′–AACACAAAGCCCACATTCCC–3′	100	58	NM_001002.3

Real-time PCR conditions were optimized as described previously (Valarmathi et al., 2008a,b; Willems et al., 2008; Bustin et al., 2009). All RT-qPCRs were performed with SsoAdvanced™ SYBR® Green Supermix in a CFX96 Touch™ Real-Time PCR Detection System (Bio-Rad Laboratories, Inc.), and C_T_ (threshold cycle) values were calculated using the CFX Manager™ software, Security Edition. The calibrator control included hCMVECs day 0 sample for vasculogenic cultures and hiPSC-ECMs day 0 sample for cardiomyogenic cultures, and the target gene expression was normalized by three non-regulated reference gene expressions, viz., GAPDH, β-ACTIN, and either G6PD or RPLP0. The expression ratio of genes was determined by applying the mathematical model previously described by Pfaffl et al. (2002).

### Immunofluorescence staining and confocal microscopy

CCC culture (vasculogenic: hCMVECs culture, hCMVECs/hMSCs co-culture; cardiomyogenic: hiPSC-ECMs culture; vascularized cardiac patch: hCMVECs/hiPSC-ECMs/hMSCs co-culture) samples were collected at day 7 or day14, and processed according to previously described protocols (Valarmathi et al., 2011), for immunostaining and phalloidin staining. The primary antibodies that were used in this study, shown in Table 1. Rhodamine labeled Ulex Europaeus Agglutinin I (1:50 in 10 mM N-2-hydroxyethylpiperazine-n'-2-ethanesulfonic acid, pH 7.5; 0.15 M NaCl; lectin, Vector Labs) was used to detect endothelial cells. DAPI (4, 6-diamidino-2-phenylindole, 100 ng/ml; Sigma-Aldrich) was used to counterstain the nuclei. Images of the stained CCCs were visualized using a confocal (Zeiss LSM 510 Meta CSLM) or a fluorescence (Olympus BX53) microscopic system. Negative controls for staining included only secondary antibodies (data not shown).

### Pharmacological assay of vascularized cardiac patch: live cell imaging with spinning disk confocal microscopy

To assess the *in vitro* functional competence of the generated vascularized cardiac patch, CCC constructs were exposed to various cardioactive pharmacological agents (0.1 to 1 μM), such as isoprenaline, clenbuterol, and diltiazem, either individually or in sequential combination. Calcium transients were recorded by live cell imaging using spinning disk confocal microscopy as described above, and the cell's chronotropic and inotropic responses were analyzed.

### Statistical analysis

The RT-qPCR experimental data were represented as mean ± standard error of the mean (mean ± SEM). The differences in expression profile (cardiomyogenic and vasculogenic markers) between control (day 0) and treated samples (day 7 or 14) were determined in-group means for statistical significance by applying ‘*Pair Wise Fixed Reallocation Randomization Test’* using Relative Expression Software Tool—384 (REST-384- version 2) (Pfaffl et al., 2002). In all cases, *p* < 0.05 were considered statistically significant.

## Results

### Expression of vasculogenic markers in prevascularized CCCs

**Figure 1:**
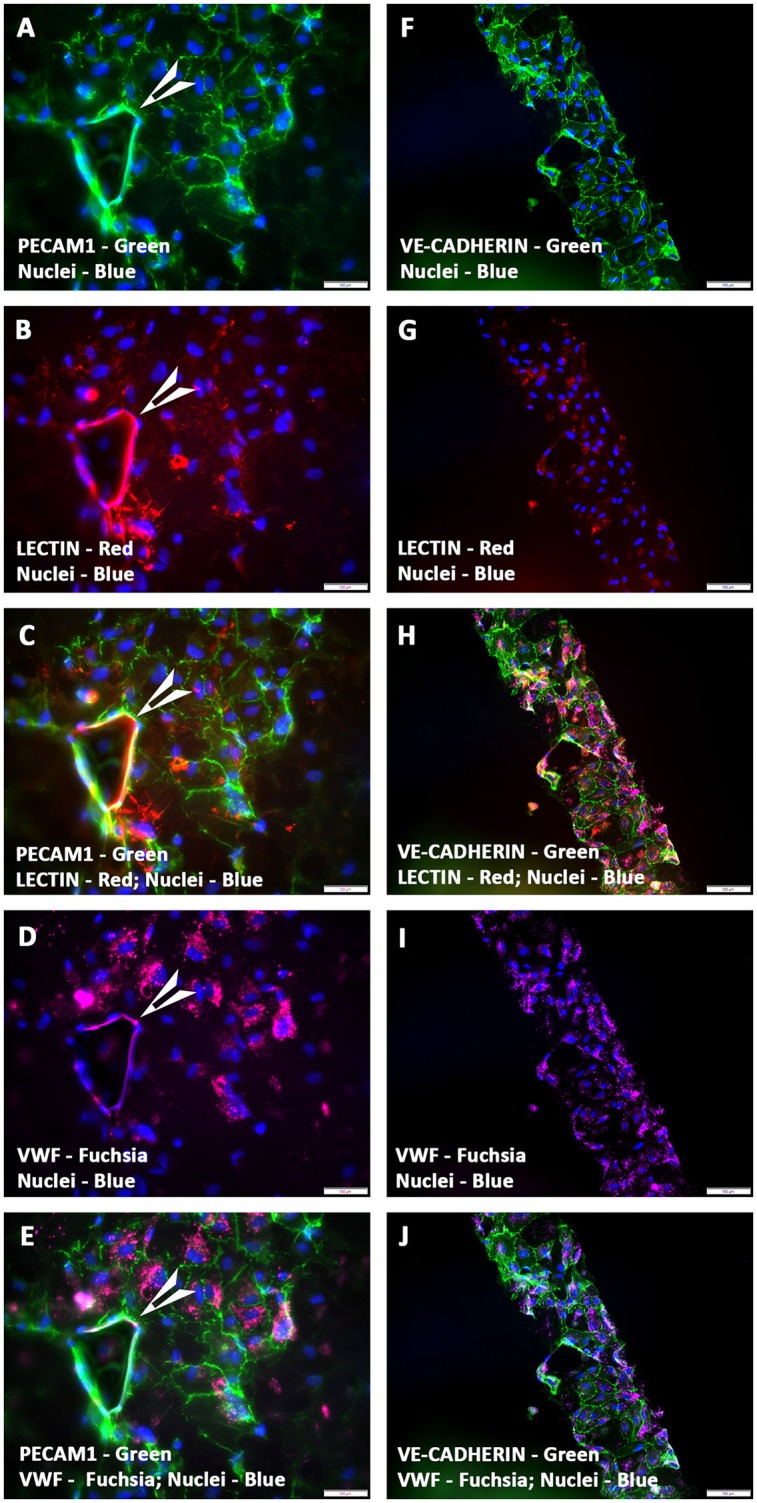
**Expression pattern of various endothelial cell markers in CCCs (hCMVECs culture) by immunofluorescence microscopy**. Localization of key endothelial cell phenotypic markers of day 14 vasculogenic hCMVECs CCC cultures demonstrated the expression of PECAM1 **(A,C,E)**, VE-cadherin **(F,H,J)**, Lectin **(B,C,G,H)**, and VWF **(D,E,I,J)**. Dual immunostainings of vasculogenic CCC cultures revealed areas of adherent sheets of polyhedral type of cells composed of mature endothelial cells, and were arranged in cobble-stone fashion **(A–J)**. These cells formed occasional nascent capillary structures with its associated lumens (arrow heads, **A–E**). In addition, these cells were organized into a cohesive array of endothelial cells, and were appeared to be retracted from the substratum and reorganized into a broad flat ribbon-like configuration **(F–J)**. These aligned and reoriented column of vascular cells were reminiscent of an *in vivo* microvessel morphogenesis **(F–J)**. Cells were also stained for nuclei (blue, DAPI). Merged images **(A–J)**. (**A–J**, scale bar 100 μm).

hCMVECs seeded onto fibronectin coated CCCs, and cultured under vasculogenic culture conditions for 14 days generated limited capillary networks. Immunostaining demonstrated these structural organizations consisted of cells that were naturally positive for a set of endothelium-associated markers, such as PECAM1, VE-Cadherin, VWF, and lectin (Figures 1A–J). This indicated that CCCs were able to provide appropriate substratum for the hCMVECs' adhesion, proliferation, and differentiation. These cells formed extensive sheets of cohesive polyhedral type of cells composed of mature endothelial cells, and were arranged in cobble-stone fashion (Figures 1A–E). Amidst these monolayered endothelium were seen nascent capillary structures with central lumens (arrow, Figures 1A–E). In addition, these hCMVECs were structured into a cohesive array of endothelial cells, and were appeared to be retracted from the substratum and reorganized into a broad flat ribbon-like configuration reminiscent of attempting vessel formation (Figures 1F–J). These aligned hCMVECs, transforming into column-like structure were evocative of earliest stages of vasculogenesis.

**Figure 2:**
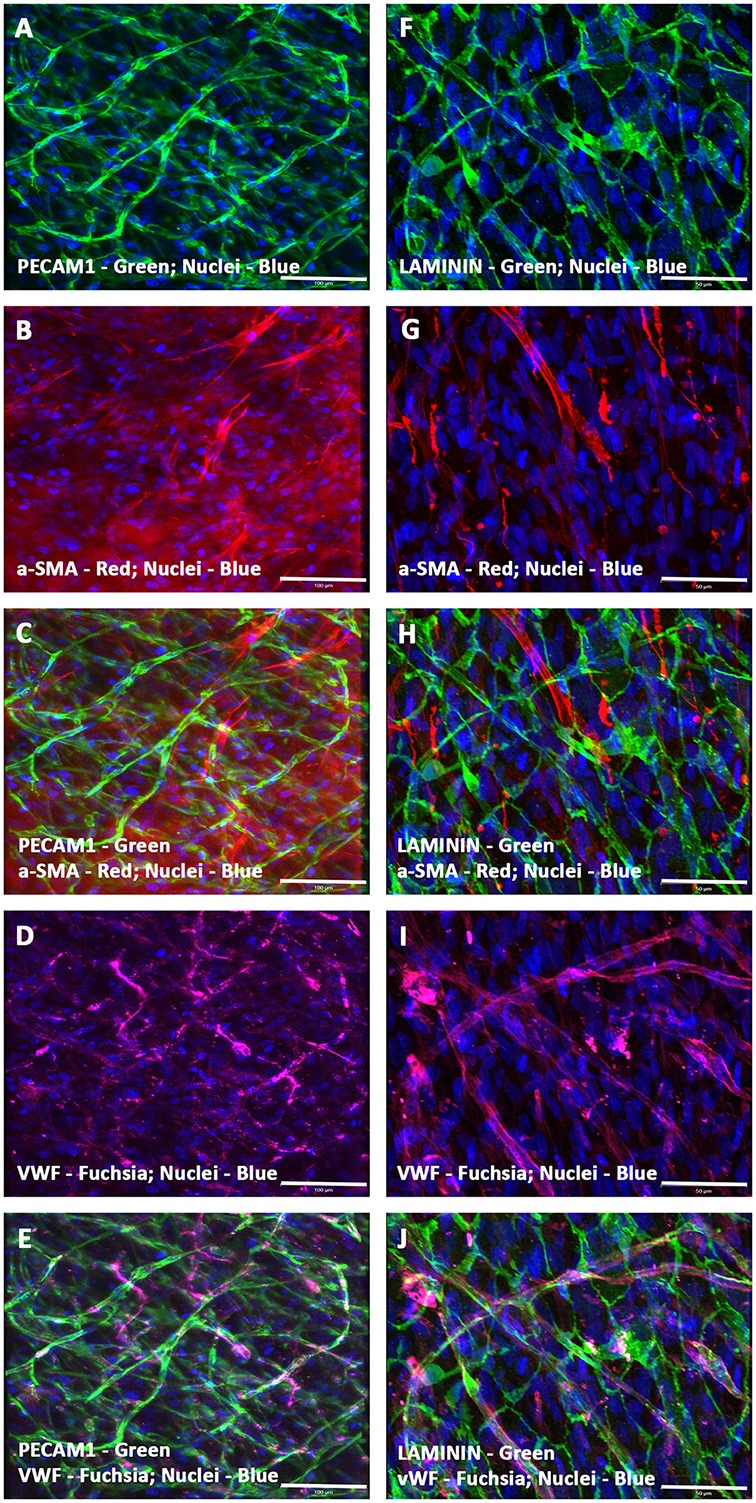
**Expression pattern of various endothelial and smooth muscle cell markers in CCCs (hCMVECs/hMSCs co-culture) by immunofluorescence microscopy**. Localization of key vascular cell phenotypic markers of day 14 vasculogenic hCMVECs/hMSCs CCC co-cultures demonstrated the expression of PECAM1 **(A,C,E)**, Laminin **(F,H,J)**, α-SMA **(B,C,G,H)**, and VWF **(D,E,I,J)**. Dual immunostainings of vasculogenic CCC co-cultures revealed alternate layers of elongated (top layer) and flattened cells (bottom layer) composed of varying degrees of mature endothelial and smooth muscle cells **(A–J)**. These cells were self-organized into remarkable plexuses of nascent microvascular structures composed of mature endothelial and/or smooth muscle cells. In addition, the linear and branching vessels were composed of laminin positive sheath-like structure covering these mixed population of vascular cells, evoking the emergence of outer thick intact basement membrane **(F,H,J)**. These capillary structures also revealed narrow and translucent central lumen. Cells were also stained for nuclei (blue, DAPI). Merged images **(A–J)**. (**A–J**, scale bar 100 μm).

On the contrary, a passage 3 pooled and almost pure population of enriched hMSCs, which were devoid of any hematopoietic stem and/or progenitor cells as well as mature endothelial cells (by FACS and MACS) when co-seeded with hCMVECs (i.e., hCMVECs/hMSCs) onto fibronectin precoated CCCs, and cultured under vasculogenic culture conditions for 14 days generated extensive plexuses of capillaries. Immunostaining and fluorescence microscopic analysis demonstrated that these micro-vascular structures were positive for a variety of markers that were not only associated with endothelial cells but also with the smooth muscle cells, such as PECAM1, VWF, α-SMA, and laminin (Figures 2A–J). By cellular adhesion and condensation these cells formed sheet of mono- and multi-layered cells. Extensive network of intertwined microvessels were seen on top of the monolayered endothelium. Beneath those transformed plexuses of the linear and arborizing vascular structures were the monolayer of cohesive endothelium, and it was apparent that there exist a continuum of transition between these two morphological layers (Figures 2A–J). The linear and branching endothelium lined vessels were surrounded by hMSC-derived α-SMA positive cells, reminiscent of the process of in situ *de novo* vasculogenesis (Figures 2B,C,G,H). And these vessels were also positive for the protein, laminin, indicating the presence of intact basement membrane material (Figures 2F,H,J).

**Figure 3:**
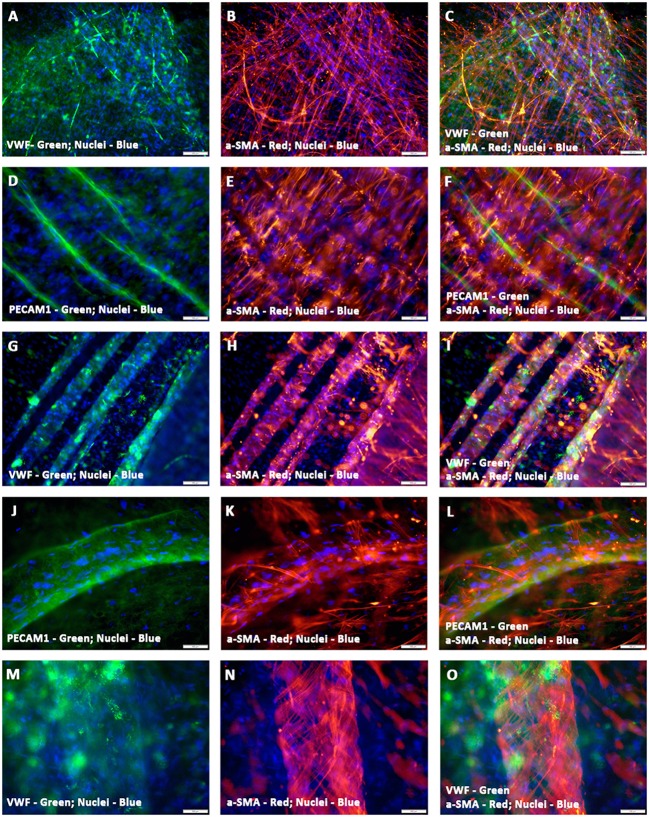
**Characterization of hCMVECs/hMSCs co-culture derived in situ *de novo* vascular structures by immunofluorescence microscopy**. Day 14 vasculogenic hCMVECs/hMSCs CCC co-cultures generated not only extensive plexuses of capillary structures lined simply by endothelial cells but also vascular structures resembling that of small to medium sized muscular arteries **(A–O)**. These thin and delicately intertwined microvascular structures expressed both endothelial and smooth muscle cell markers, VWF and α-SMA, respectively, and revealed apparent areas of co-localizations **(A–C)**. The PECAM1 positive endothelial cells formed a linear solid cord-like structure, and were uniformly surrounded by the α-SMA positive smooth muscle cells, and these α- SMA positive cells were oriented in a direction that was perpendicular to the direction of linearly assembled endothelial cells **(D–F)**. Further morphogenesis of these endothelium lined tubular structures illustrated the dynamic process of evolving tunica media, the hMSC-derived α-SMA positive cells were wrapping around the entire circumference of these endothelial outgrowth **(G–I)**. Subsequent stages of morphogenesis revealed emergence of greater caliber vessels, having sleeve-like outer smooth muscle cells encasing the endothelium lined tubular structure **(J–O)**. Merged images **(A–O)**. (**A–O**, scale bar 100 μm).

Furthermore, the hCMVECs/hMSCs co-cultures demonstrated thin and delicately intertwined microvascular structures expressing both endothelial and smooth muscle cell markers, VWF and α-SMA, respectively, and revealed apparent areas of co-localizations (Figures 3A–C). Apart from this day 14 culture also displayed larger caliber vascular structures, such as medium sized muscular arteries (Figures 3D–O). The morphogenesis of these medium-sized muscular arteries were evident, initially, the α-SMA positive cells were able to migrate toward the linear array of endothelium lined tubular structures, and were able to align in a perpendicular fashion to the long axis of these parallelly arranged tubular structure or solid cords of coalesced cells (Figures 3D–F). Further progressive morphogenesis revealed that these α-SMA positive cells were able to wrap around these tubular structure in a concentric fashion (Figures 3G–I), and formed an outer continuous sleeve-like structure (Figures 3J–O). These vessels revealed strong expression of mature and terminally differentiated endothelial and smooth muscle cell markers, viz., VWF and α-SMA, respectively. Hence, suggestive of mature vascular phenotype. These snapshots may be reminiscent of many aspects of *in vivo* morphogenesis of medium- and/or large-sized muscular arteries.

### Evaluation of CCC vascular structures for LDL uptake

**Figure 4:**
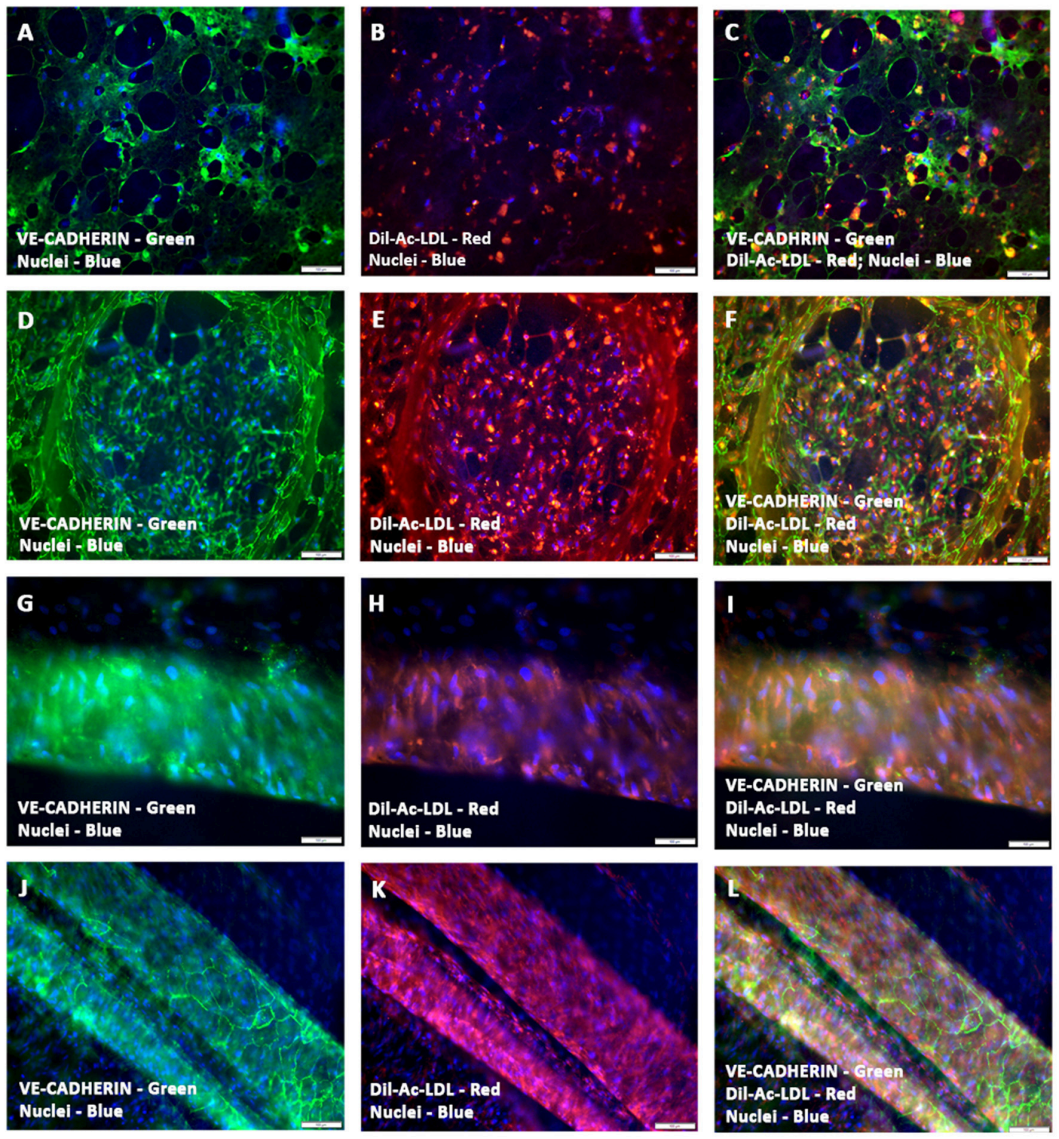
**Functional characterization of prevascularized CCCs by Dil-Ac-LDL uptake assay**. hCMVECs cultured or hCMVECs/hMSCs co-cultured onto CCCs in vasculogenic culture conditions were incubated with 10 μg/ml of Dil-Ac-LDL for 4 to 6 h. Fluorescence microscopic analysis of hCMVECs only CCCs revealed abundant punctate perinuclear bright red fluorescence of differentiated and matured endothelial cells (day 7, **B,C**; day 14, **E,F**). These labeled vascular cells were organized into a delicate network of capillaries **(A–C)** or into a discrete cluster **(D–F)**. Similarly, fluorescence microscopic analysis of hCMVECs/hMSCs co-cultured CCCs demonstrated typical abundant punctate perinuclear bright red fluorescence of the differentiated and matured endothelial cells (day 7, **H,I**; day 14, **K,L**). These Dil-labeled endothelial cells were structured into solid cohesive columns of VE-cadherin positive cells, mimicking functionally competent larger caliber vessels **(J–L)**. Cells were also stained for nuclei (blue, DAPI) and endothelial cells (green, VE-cadherin, **A,C,D,F,G,I, J,L**). Merged Images **(A–L)**. (**A–L**, scale bar 100 μm).

A functional approach was undertaken to characterize the phenotypic nature of these preformed vessels, by measuring the uptake of the fluorescent compound, Dil-Ac-LDL. Immunofluorescence analysis of Dil-Ac-LDL stained prevascularized CCCs showed strong uptake of LDL from the culture medium. The sheets and clusters of endothelial cells (Figures 4A–F) as well as endothelium lined tubular structures of various calibers (Figures 4G–L), were intensely positive for the staining, indicating the high metabolism of the protein, Ac-LDL, in these prevascularized CCCs. Fluorescence signals were largely punctate and were perinuclear in arrangement. In hCMVECs/hMSCs CCCs, the hMSC-derived smooth muscle cells were discriminated from hCMVECs by their lack of detectable Dil fluorescence signal, and their presence can be easily detected as DAPI-stained surrounding bare nuclei (Figures 4G–L). The Ac-LDL update and its metabolism were apparent in day 7 (hCMVECs culture, Figures 4A–C; hCMVECs/hMSCs co-culture, Figures 4G–I) and day 14 (hCMVECs culture, Figures 4D–F; hCMVECs/hMSCs co-culture, Figures 4J–L), with an enhanced level of uptake seen at day 14, visually. Figures 4J–L were showing typical medium-sized muscular tube-like vessels that contain obvious bright-red stained solid cord of endothelial cells covering presumably the interior surface.

### SEM analysis of prevascularized CCCs

**Figure 5:**
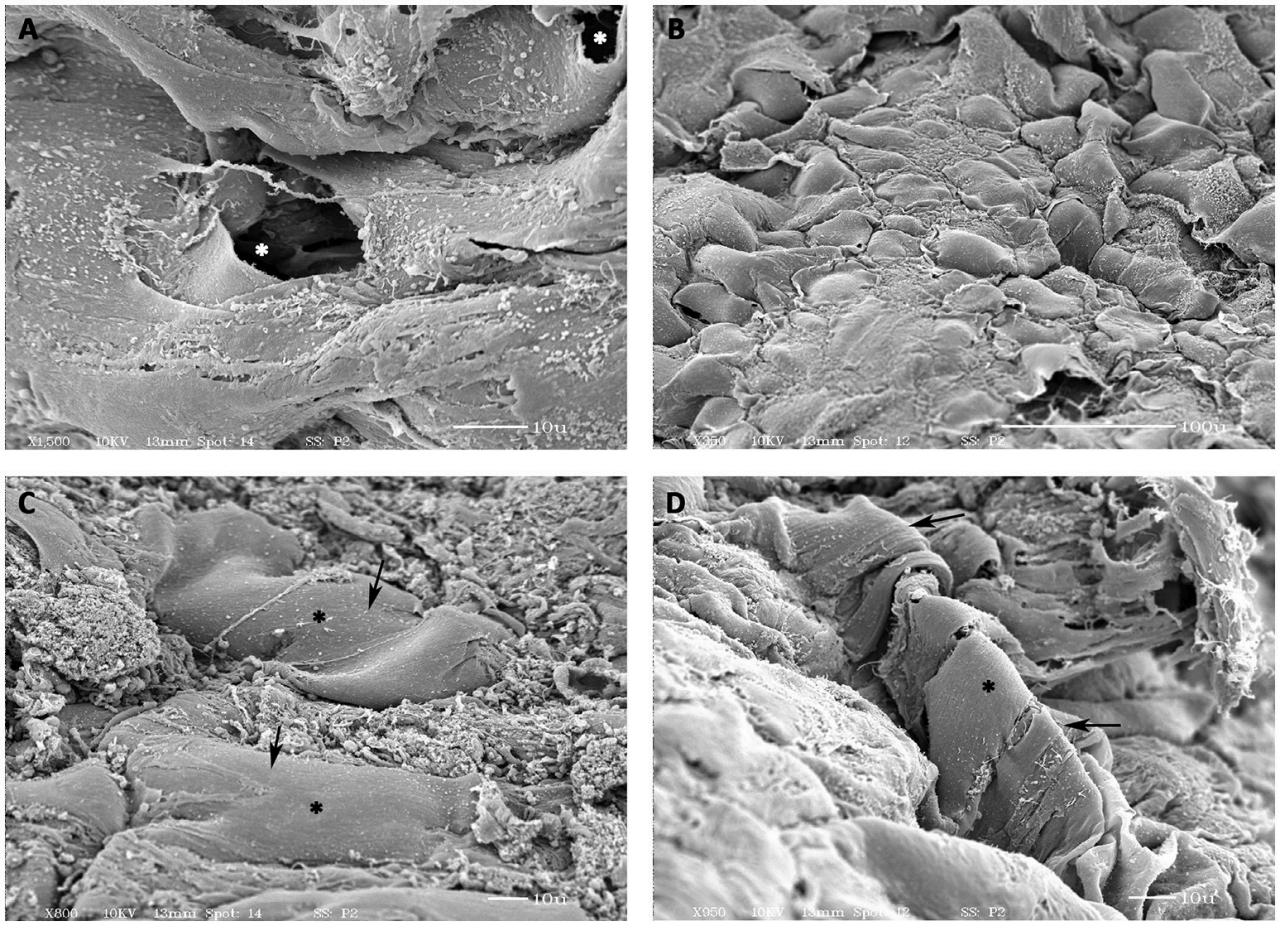
**Scanning electron microscopic (SEM) analysis of prevascularized CCCs**. SEM analysis of day 14 vasculogenic hCMVECs/hMSCs CCC cultures showed the typical transverse capillary-like structure with its central lumen (white, asterisks, **A**), composed of flattened layer of differentiated endothelial cells. In addition, these differentiated cells revealed foci of flattened cells (cobble-stone appearance) in juxtaposition with foci of evolving cellular retraction and transformation **(B)**. Besides, presence of smooth-walled tube-like structures with its attached smooth muscle cells were evident (black arrows, **C,D**). Multiple smooth muscle-like cells were obviously in the process of wrapping around these tube-like structures (black asterisks, **D**). Some of those cylindrical structures revealed the presence of evolving patent lumens or cavernous spaces (white asterisks, **A**). (**A,C,D**, scale bar 10 μm; **B**, scale bar 100 μm).

SEM analysis of the day 14 hCMVECs/hMSCs based prevascularized CCCs revealed typical transverse capillary structures lined by two to three differentiated endothelial cells and its associated lumens (white asterisk, Figure 5A). In addition, these differentiating cells showed foci of flattened cells arranged in cobble-stone fashion and were in juxtaposition with foci of cellular retraction and transformation (Figure 5B). Furthermore, areas of elongated and convoluted multilayered tube-like structures with attached hMSC-derived smooth muscle cell were seen (black asterisks, Figures 5C,D). It was clearly evident that these smooth muscle cells were attempting to wrap around these tubular structures (black asterisks, Figure 5D). These endothelium and smooth muscle layered tubular structures revealed emerging interconnected conduits.

### Transmission electron microscopic (TEM) analysis of prevascularized CCCs

**Figure 6:**
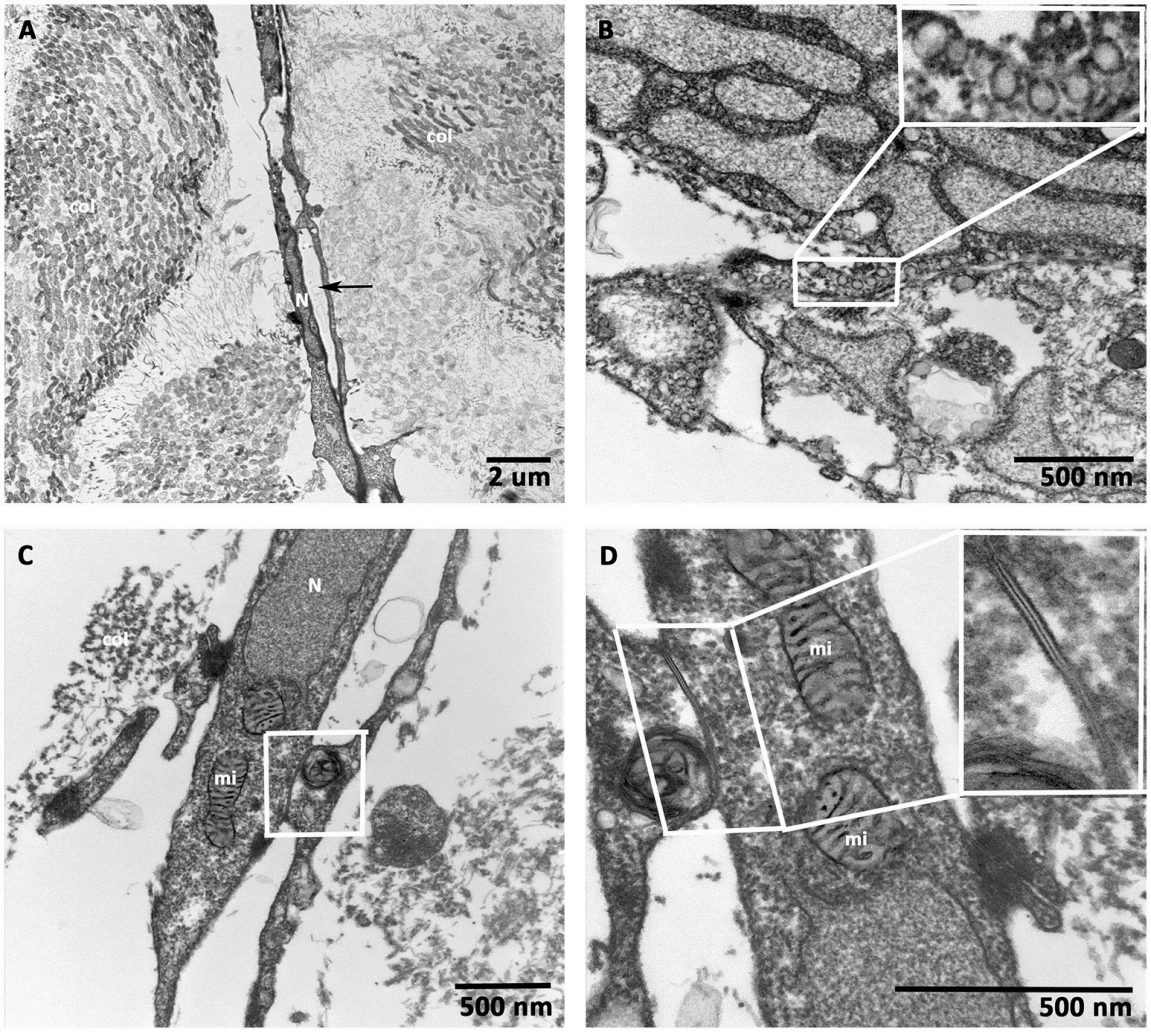
**Transmission electron microscopic (TEM) analysis of prevascularized CCCs**. TEM analysis of day 14 vasculogenic hCMVECs/hMSCs CCC cultures revealed an elongated vessel-like structure composed of endothelial cells, which were arranged on either side of a linear slit-like space (black arrows, **A**). Randomly oriented bundles of collagen fibrils were evident on either side of this elongated capillary. Note the most characteristic feature of endothelial cell, small membrane-bound vesicles, resembled a little flask, or caveola, and occupied up to a quarter of the endothelial cells (**B**, insert). The interdigitating endothelial cells showed the junctional complex (**C**, lower magnification). The typical adherent junction could be visualized between two overlapping endothelial cell processes (**D**, higher magnification, insert). (**A**, scale bar 2 μm; **B–D**, scale bar 500 nm).

hCMVEC-derived continuous capillary with the complete endothelium was seen in Figure 6A. The juxtaposed endothelial cell were overlapping. They were in contact and tethered to each other by both tight and adhering junctions as seen in (inserts, Figures 6C,D). A barrier that may impedes intercellular transport. Numerous caveolae were visible close to the cell surfaces and immediately beneath it, and small transport vesicles were also seen in abundant in the cytoplasm of these endothelial cells, as shown in (Figure 6B). The complete endothelial part of the capillary was surrounded by a continuous basal lamina, embracing the endothelium with its associated cells. In addition, numerous endothelial cell-specific storage vesicles and the electron-dense bodies were seen in Figure 6A.

### Expression of cardiomyogenic markers in hiPSC-ECMs and hiPSC-ECMs/hMSCs cultures

#### Characterization of hiPSC-ECMs CCCs

**Figure 7:**
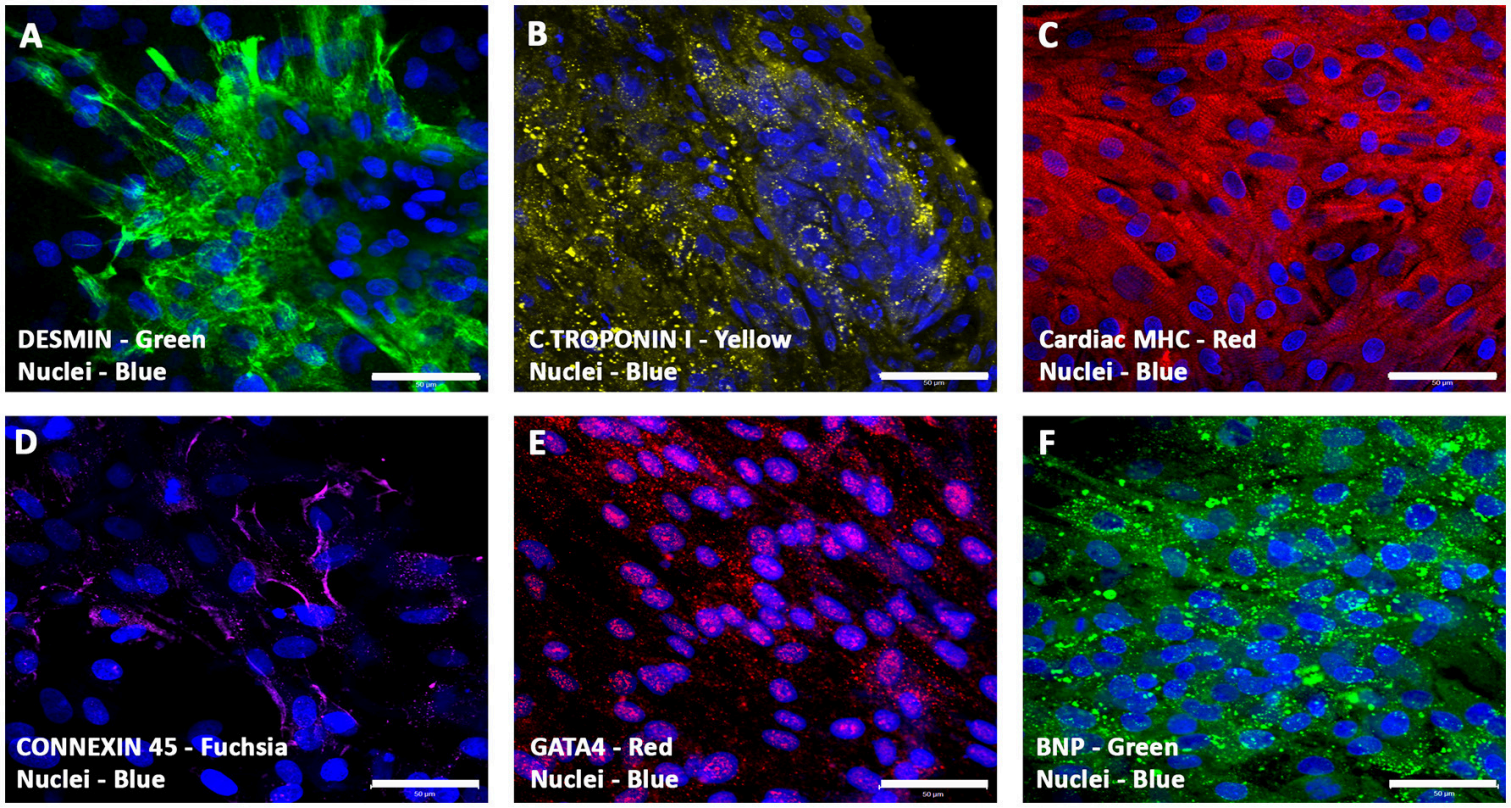
**Expression pattern of various cardiomyogenic markers in CCCs (hiPSC-ECMs culture) by confocal scanning laser microscopy**. Localization of key cardiac myocyte phenotypic markers of day 14 hiPSC-ECMs CCC cultures in myocyte maintenance medium (maintenance medium, iCell) demonstrated the expression of cardiac specific structural, contractile, and functional proteins: muscle-specific intermediate filament, desmin **(A)**, cardiac troponin, cTnI **(B)**, cardiac myosin heavy chains, α/β-MHC **(C)**, gap junction proteins, connexon, Cx45 **(D)**, transcription factor, GATA4 **(E)**, and peptide hormone, BNP **(F)**. Immunostaining of hiPSC-ECMs CCC cultures in myocyte medium showed foci of typical elongated strap-like cells with periodic cross-striations, and by means of cellular condensation these myocytes were organized into multilayered functional syncytium **(A–F)**. In some foci, these myocytes were aligned and overlapping with each other in an orderly manner **(A–F)**. Nuclei of these cells were large and either oval or round in appearance, and were centrally positioned. Cells were also stained for nuclei (blue, DAPI). Merged images **(A–F)**. (**A–F**, scale bar 100 μm).

Immunostaining and confocal microscopic analysis of differentiating cells using a set of cardiac myocyte differentiation markers, especially, the cardiac-related structural and functional proteins were carried out. The hiPSC-ECMs containing CCCs revealed that these cells were positive for a battery of myocyte specific markers, such as desmin, cardiac troponin I (cTnI), cardiac myosin heavy chain (α/β-MHC), connexin 45 (Cx45), GATA-binding protein 4 (Gata4), and brain natriuretic peptide (BNP). Typical staining patterns expressed by cell in hiPSC-ECMs only cultures were shown in Figures 7A–F). Analysis of day 14 hiPSC-ECMs CCCs displayed multiple foci of elongated strap-like cells with periodic cross-striations and/or polyhedral type of cells, and by means of cellular condensation these myocytes were organized into multilayered functional syncytium. Majority of the cells exhibited uniform alignment, and were in registry. The myocytes demonstrated the presence of evolving sarcomeric units that were positive for various cytoskeletal filamental proteins, myosin and desmin (Figures 7A,C), as well as positive for the gap junction protein, connexon 45 (Figure 7D), suggestive of embryonic cardiac myocyte phenotype.

#### Cellular morphology and contractility (hiPSC-ECMs/GFP-hMSCs CCCs)

**Figure 8:**
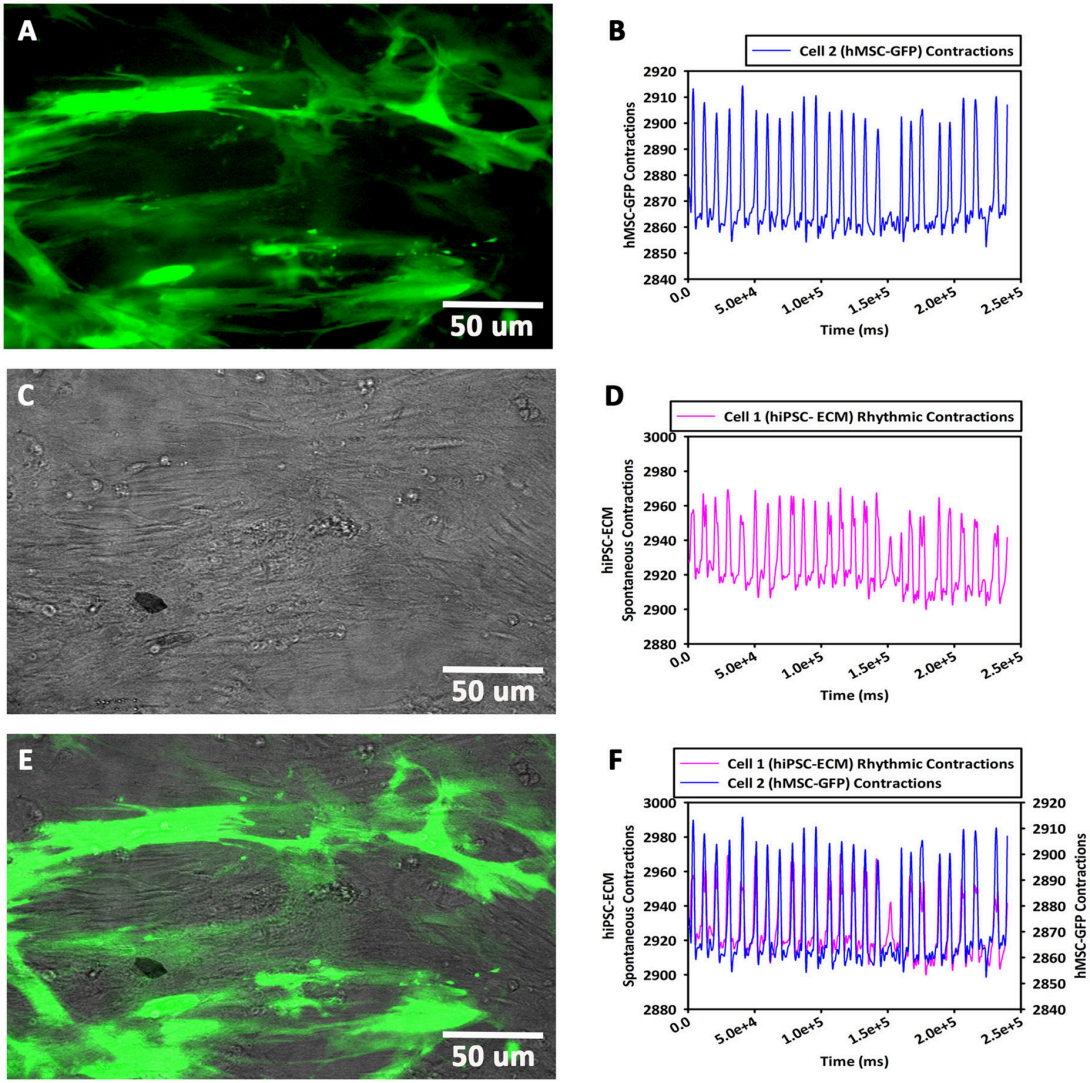
**Contractility assay of CCCs (hiPSC-ECMs/GFP-hMSCs co-culture) by live cell imaging with spinning disk confocal microscopy**. In hiPSC-ECMs/GFP-hMSCs co-culture, the green fluorescent protein tagged hMSCs (GFP-hMSCs) were tethered to hiPSC-ECMs and were mechanically coupled to each other. The pattern and frequency of contractions of the coupled GFP-hMSCs were principally similar to the pattern and frequency of hiPSC-ECMs contractions. The frequency of cellular movement of hMSC was in principle determined by hiPSC-ECMs contractile frequency. Typical fluorescence image of GFP-hMSCs attached to hiPSC-ECMs, shown in **(A)**. The mechanically coupled hMSCs were stretched and assumed prolate ellipsoid type of morphology. The pattern and frequency of GFP-hMSC cellular movement and contraction, shown in **(B)**. Typical phase contrast image of GFP-hMSCs tethered to hiPSC-ECMs, shown in **(C)**. The pattern and frequency of hiPSC-ECM's spontaneous and rhythmic contractions, shown in **(D)**. Superimposition of fluorescent and phase contract images, illustrating relative locations of GFP-hMSCs and hiPSC-ECMs in this CCC co-culture, shown in **(E)**. Comparison of GFP-hMSC pattern of cellular movement with respect to hiPSC-ECM cellular contractility revealed that the frequency of movement of hMSC was principally in synchrony and determined by the frequency of hiPSC-ECM contractions, shown in **(F)**. (**A,C,E**, scale bar 50 μm).

Within 24 h, the GFP-hMSCs, which were in co-culture with hiPSC-ECMs showed cellular morphological changes, and were able to couple with neighboring hiPSC-ECMs. The mechanically coupled cells, viz., GFP-hMSCs and hiPSC-ECMs were able to undergo synchronized cellular contractions. Observation of day 14 culture demonstrated that GFP-hMSCs were not only mechanically coupled to the neighboring hiPSC-ECMs but also revealed morphological deformation, in essence exerted by the spontaneously contracting juxtaposed and coupled hiPSC-ECMs (Figures 8A–F). In this process of cyclical stretching and/or deformation GFP-hMSCs expressed phenotypic changes, and resembling that of a prolate ellipsoid (Figure 8A). Whereas, the control CCCs cultures that consisted solely hMSCs in the same culture medium conditions expressed the cellular phenotype of an oblate spheroid. hMSCs that were in non-co-culture situations (negative controls) and/or not in association with hiPSC-ECMs (in co-culture) displayed random non-recurring changes that were related in general with common cellular migration and movements.

#### Cellular calcium flux (hiPSC-ECMs/GFP-hMSCs CCCs)

**Figure 9:**
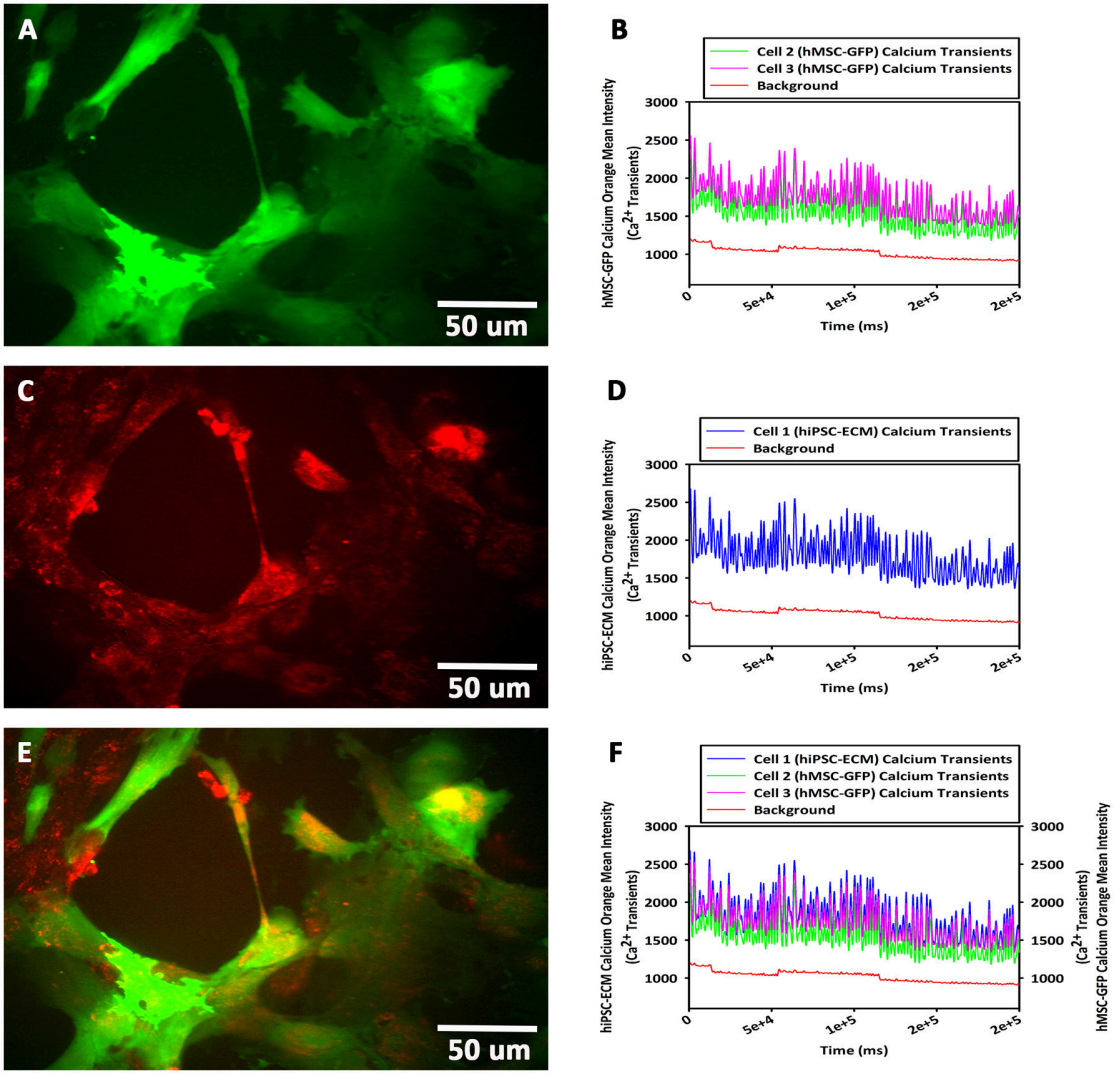
**Calcium flux assay of CCCs (hiPSC-ECMs/GFP-hMSCs co-culture) by live cell imaging with spinning disk confocal microscopy**. Green fluorescent protein tagged hMSCs (GFP-hMSCs) were mechanically coupled with spontaneously contracting hiPSC-ECMs and revealed intracellular calcium flux with similar frequency but relatively lesser amplitude. GFP-hMSCs were labeled with Calcium Orange, the calcium indicator, and imaged in the green channel using live cell confocal microscope, shown in **(A)**. The pattern of GFP-hMSCs intracellular calcium flux measured in the red channel for Calcium Orange, shown in **(B)**. hiPSC-ECMs and GFP-hMSCs were labeled with Calcium Orange and imaged in the red channel, shown in **(C)**. The pattern of spontaneously and rhythmically contracting hiPSC-ECM intracellular calcium flux measured in the red channel for Calcium Orange, shown in **(D)**. Superimposition of green channel image of GFP-hMSCs labeled with the calcium indicator, Calcium Orange, and the red channel image of hiPSC-ECMs and GFP-hMSCs labeled with Calcium Orange in this CCC co-culture, shown in **(E)**. Assessment of the pattern of GFP-hMSCs calcium flux with the pattern of contractile hiPSC-ECM displayed that the intracellular oscillation of calcium of hMSCs were fundamentally the same as that of hiPSC-ECM but with relatively lesser spikes, shown in **(F)**. (**A,C,E**, scale bar 50 μm).

To study the cellular calcium flux, GFP-hMSCs were co-cultured with spontaneously and rhythmically beating hiPSC-ECMs. GFP-hMSCs, which were mechanically associated with hiPSC-ECMs demonstrated intracellular calcium spikes typified by repeated increase of the cytosolic Ca^2+^ concentration and a subsequent removal of Ca^2+^ as depicted (Figures 9A,B). When imaged over a period of 5 min or greater, the GFP-hMSC, which was mechanically linked to an electrically paced juxtaposed hiPSC-ECM (Figures 9C,D) revealed calcium oscillations/spikes, which were essentially similar in pattern and frequency to that of the hiPSC-ECM (Figures 4E,F). When two individual hMSCs, which were not directly in contact with each other, but were coupled with the same individual electrically paced hiPSC-ECM, the pattern and frequency of those two hMSCs' intracellular calcium spikes were virtually comparable (Figures 9A,B). The temporal and spatial pattern of the intracellular calcium oscillations in these two discrete hMSCs were in essence similar to that observed in the case of hiPSC-ECM (Figures 9E,F). In addition, the control hMSCs, which were in non-co-cultured conditions revealed negligible intracellular calcium oscillations (Figures 9B,D,F, the bottom red line).

### Ultrastructural morphology of CCCs (hMSCs, hiPSC-ECMs, and hiPSC-ECMs/hMSCs)

**Figure 10:**
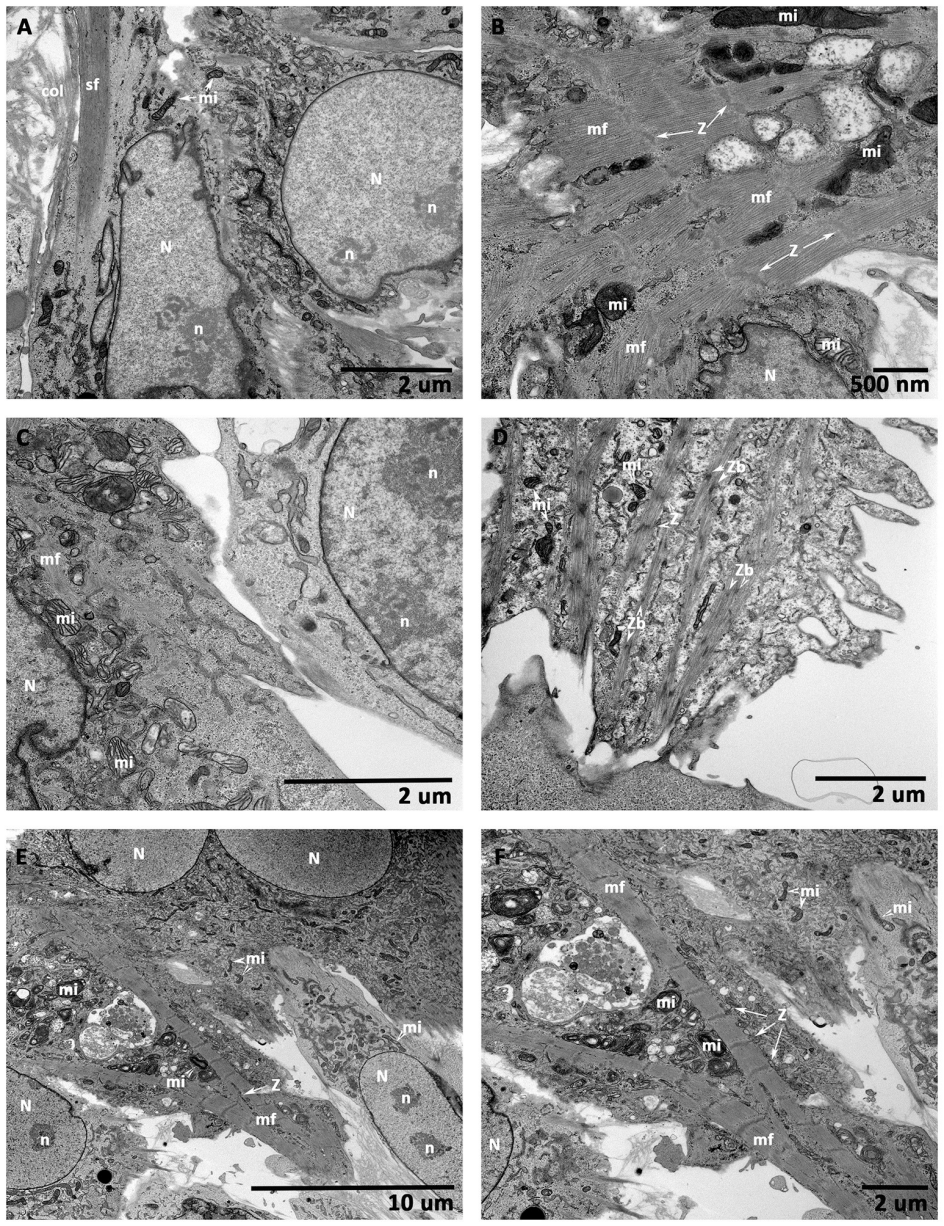
**Transmission electron microscopic (TEM) analysis of CCCs (hMSCs culture, hiPSC-ECMs culture, and hiPSC-ECMs/hMSCs co-cultures)**. hMSCs cultured on CCCs in myocyte medium showed euchromatic nuclei (N) with single to multiple nucleoli (n), and the cytoplasm was characterized by numerous random mitochondria and widespread endoplasmic reticulum, the ultrastructural characteristics of an active and immature cells **(A)**. Besides, the hMSCs cytoskeleton exhibited bundles of actin stress fibers (sf) predominantly confined toward the peripheral cytoplasm. When hiPSC-ECMs cultured on CCCs under the same culture conditions revealed active euchromatic nucleus (N). The cytoplasm revealed myofibrils (mf), perinuclear early sarcomeric units, Z-discs (Z), and few mitochondria (mi) interspersed between the myofibrils along with glycogen particles **(B)**. Whereas, the hiPSC-ECMs/hMSCs CCC co-cultures revealed that the differentiating hMSCs were in juxtaposition and tethered by numerous focal adhesions localized along the cellular margins of adjacent hiPSC-ECMs. The myofibrils of the coupled myocytes frequently exhibited a number of discontinuous and thickened Z discs as well as indistinct or absent M bands. Disorganized actin and myosin filaments not assembled into myofibrils were also seen in the cytoplasm **(C)**. Some disorganized thin filaments were continuous with myofibrils, and were often attached to the sarcolemma by focal adhesions **(C)**. Occasional developing myocytes illustrated typical imperfections of the myofibrillar organization, characterized by discontinuous and/or widened Z discs, indistinct H zones, free floating myosin filaments, as well as large number of glycogen granules; apparently mimicking like a specialized sino-atrial type of cells; besides, there were evidently numerous ovoid or circular electron-dense bodies, the Z-bodies (Zbs), composed of short sarcomeric units of α-actinin, the Z-bands were created by the fusion of these precursor Z-bodies **(D)**. In addition, some differentiating myocytes showed branching and strand-like myofibrils with regular Z disks and distinct A and I bands. The myofibrils were stretched through the entire length of the cells, with scattered pleomorphic mitochondria and tubules of sarcoplasmic reticulum. Glycogen granules were also widespread throughout the remainder of the cell. These myocytes were appeared to be fused with their adjacent hMSCs, with imperceptible demarcation of their cellular boundaries **(E,F)**. (**A,C,D,F**, scale bar 2 μm; **B**, scale bar 500 nm; **E**, scale bar 10 μm).

TEM analysis of an hMSCs cultured on CCCs in myocyte medium appeared to be characteristic of an immature and active cells (Figure 10A). These cells revealed relatively large centrally positioned nucleus with extensively opened out chromatin and varied number of nucleoli. In addition, there were numerous randomly positioned mitochondria as well as network of endoplasmic reticulum. The marginal cytoplasm contained discrete bundles of actin stress fibers (Figure 10A). When hiPSC-ECMs cultured on CCCs under similar culture conditions demonstrated active euchromatic nucleus. The cytoplasm revealed myofibrils, perinuclear early sarcomeric units, Z-discs, and few mitochondria interposed between the myofibrils along with glycogen particles (Figure 10B).

On the other hand, the hiPSCs/hMSCs containing CCCs showed characteristic ultrastructural morphology of co-differentiating cardiac myocytes. Majority of cells showed centrally positioned oval to elongated nucleus and perinuclear cytoplasm consisting of evolving myofibrillar organization with prominent sarcomeres. In some cases, the myofibrils were stretched through the entire length of the cells, with scattered pleomorphic mitochondria and tubules of sarcoplasmic reticulum. These mitochondria exhibited densely packed cristae, and few of them showed inter myofibrillar predisposition. Glycogen particles were also noticeable and were widespread throughout the remainder of the cell. The energy storing, energy releasing, and energy recapturing structures and organelles, such as mitochondria and glycogen granules, were obviously visible and abutting the myofibrillar organization, where the energy was utilized for contraction. In addition, cross-banded pattern apparently seen in the cytoplasm reflected the arrangement, in register, of the myofibrils. And the banded pattern of the myofibrils reflected the arrangement of myofilaments. The intensely stained A-bands (A—anisotropic), due to myosin filaments, and the less intensely stained I-bands (I—isotropic), composed of actin filaments, were visualized. These myocytes were appeared to be fused with their adjacent hMSCs, with imperceptible demarcation of their cellular margins (Figures 10C,E,F).

Occasional developing myocytes illustrated typical imperfections of the myofibrillar organization, characterized by discontinuous and/or widened Z discs, indistinct H zones, free floating myosin filaments, as well as large number of glycogen granules; apparently mimicking like a specialized sino-atrial type of cells (Figure 10D).

### mRNA analysis of CCCs for vasculogenic and cardiomyogenic markers

**Figure 11:**
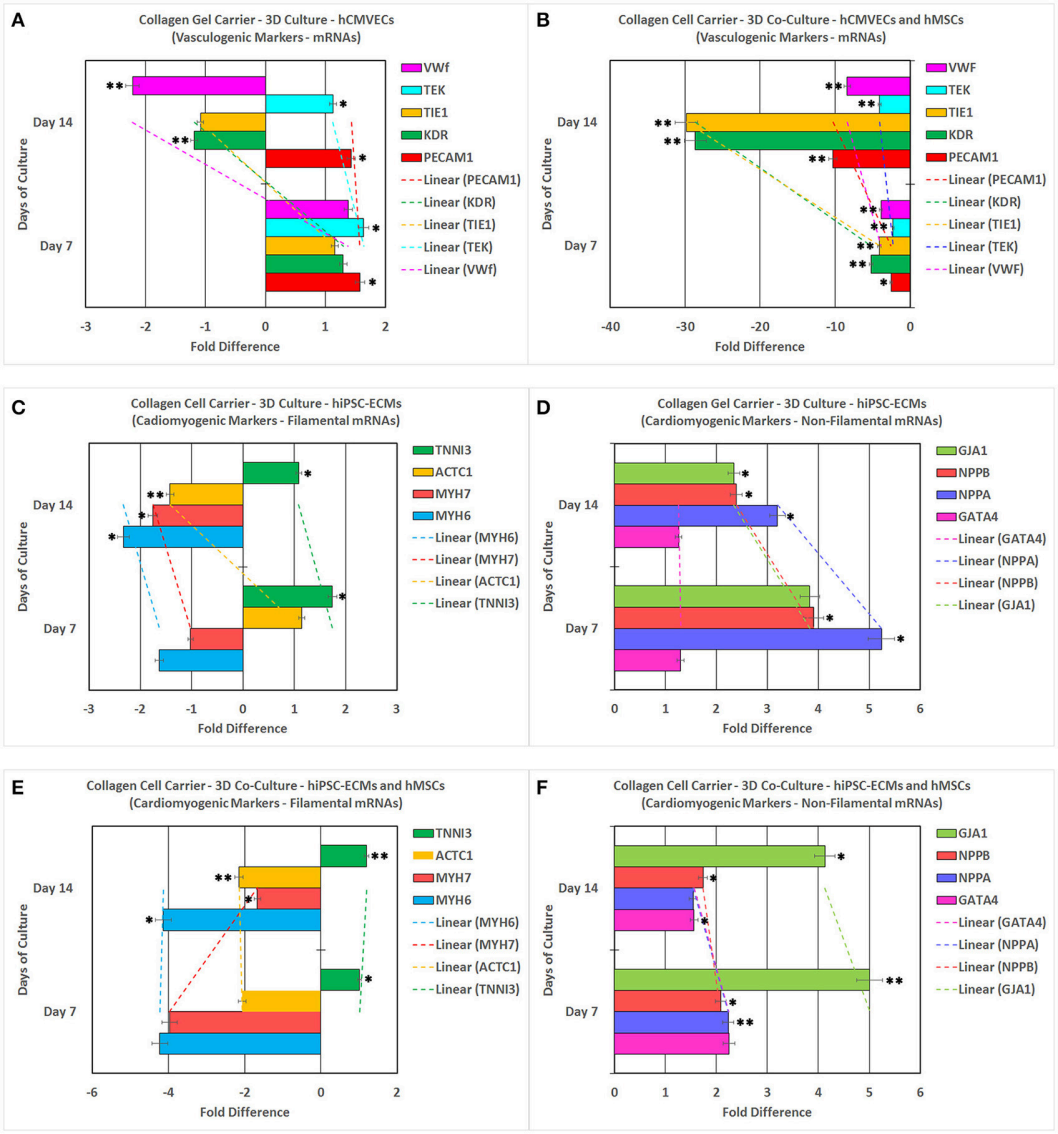
**Reverse transcription-quantitative real-time polymerase chain reaction (RT-qPCR) analysis of various key vasculogenic and cardiomyogenic markers**. PECAM1 (platelet and endothelial cell adhesion molecule 1), KDR (kinase insert domain receptor, a type III receptor tyrosine kinase), TIE1 (tyrosine kinase with immunoglobulin-like and EGF-like domains 1), TEK (TEK tyrosine kinase, endothelial), and VWF (von Willebrand factor) expression (abscissa) as a function of time (ordinate). hCMVECs cultured onto CCCs in complete microvascular endothelial cell growth medium **(A)**. hCMVECs/hMSCs co-cultured onto CCCs in complete microvascular growth medium **(B)**. Similarly, MYH6 (myosin, heavy chain 6, cardiac muscle, alpha), MYH7 (myosin, heavy chain 7, cardiac muscle, beta), ACTC1 (actin, alpha, cardiac muscle 1), TNNI3 (troponin I3, cardiac type), GATA4 (GATA binding protein 4), NPPA (natriuretic peptide A), NPPB (natriuretic peptide B), and GJA1 (gap junction protein, alpha 1) expression (abscissa) as a function of time (ordinate). hiPSC-ECMs cultured onto CCCs in complete myocyte medium **(C,D)**. hiPSC-ECMs/hMSCs co-cultured onto CCCs in complete myocyte medium **(E,F)**. The calibrator control included hCMVECs day 0 sample for vasculogenic cultures and hiPSC-ECMs day 0 sample for cardiomyogenic cultures, and the target gene expression was normalized by three non-regulated reference gene expressions, viz., GAPDH, β-ACTIN, and either G6PD or RPLP0. The expression ratio (abscissa) was calculated using the Relative Expression Software Tool–384 (REST-384 - version 2). The values were means ± standard errors for three independent cultures (*n* = 3), ^*^p < 0.05; ^**^p < 0.001.

hCMVECs cultured on CCCs in vasculogenic medium constitutively expressed mRNA transcripts coding for significant vasculogenic markers (Figures 11A,B). The PECAM1, KDR, TIE1, TEK, and VWF showed initial upregulation around day 7 followed by sustained down regulation around day 14 (Figure 11A). On the other hand, when hCMVECs/hMSCs co-cultured on CCCs in vasculogenic medium, the transcription level of PECAM1, KDR, TIE1, TEK, and VWF depicted an initial slight down regulation around day 7 followed by progressive down regulation around day 14 (Figure 11B). The KDR and TIE1 showed a remarkable level of down regulation during day 14.

hiPSC-ECMs cultured in myogenic culture conditions revealed constitutive expression of cardiomyogenic markers (Figures 11C,D). The structural and contractile filamental associated genes, MYH6 and MYH7, showed initial down regulation around day 7 with concurrent progressive gradual downregulation of these transcripts over the observed time period, around 14 days (Figure 11C). Whereas, the other filamental genes, ACTC1 and TNNI3 showed initial upregulation followed by sustained down regulation of their transcripts (Figure 11C). Besides, in hiPSC-ECMs cultures, the transcript levels of non-filamental associated genes, GATA4, NPPA, NPPB, and GJA1 demonstrated an initial upregulation around day 7, with a noticeable kinetics of gradual downregulation of these transcripts over the successive experimental time point, i.e., around day 14 (Figure 11D).

In contrast, the hiPSC-ECMs/hMSCs that were co-cultured in myogenic culture conditions revealed subtle differences in their pattern of expression, especially the structural and contractile filamental genes. hiPSC-ECMs/hMSCs co-cultured in myogenic medium expressed the cardiomyocyte differentiation related marker transcripts consistently. The structural and contractile filamental associated genes, MYH6, MYH7, and ACTC1 showed initial downregulation around day 7, and expression level of these marker transcripts were either sustained at the same level (MYH6, ACTC1) or upregulated (MYH7) during successive time point, the day 14. And on the other hand, the TNNI3 showed a gradual upregulation from day 7 to day 14 (Figure 11E).

In addition, in hiPSCs/hMSCs co-cultures, the transcript levels of GATA4, NPPA, NPPB, and GJA1 showed initial notable upregulation of these marker transcripts around day 7 and there exist a continual gradual downregulation of these transcript over the observed consecutive time point, i.e., around day 14 (Figure 11F). On the contrary, the connexon gap junction gene, GJA1 showed sustained elevated levels of expression on both day 7 and day 14.

Finally, the observed patterns of differential gene expressions, in these disparate vasculogenic and myogenic culture conditions, were not only indicative of cell-cell interactions but also suggestive of cell-matrix interactions, which ultimately led to the modulation of vasculogenic and cardiomyogenic gene expressions in this milieu, and consequently, the differentiation potentials of these cells on the CCC scaffold.

### Characterization of vascularized cardiac patch (hCMVECs/hiPSC-ECMs/hMSCs co-culture)

**Figure 12:**
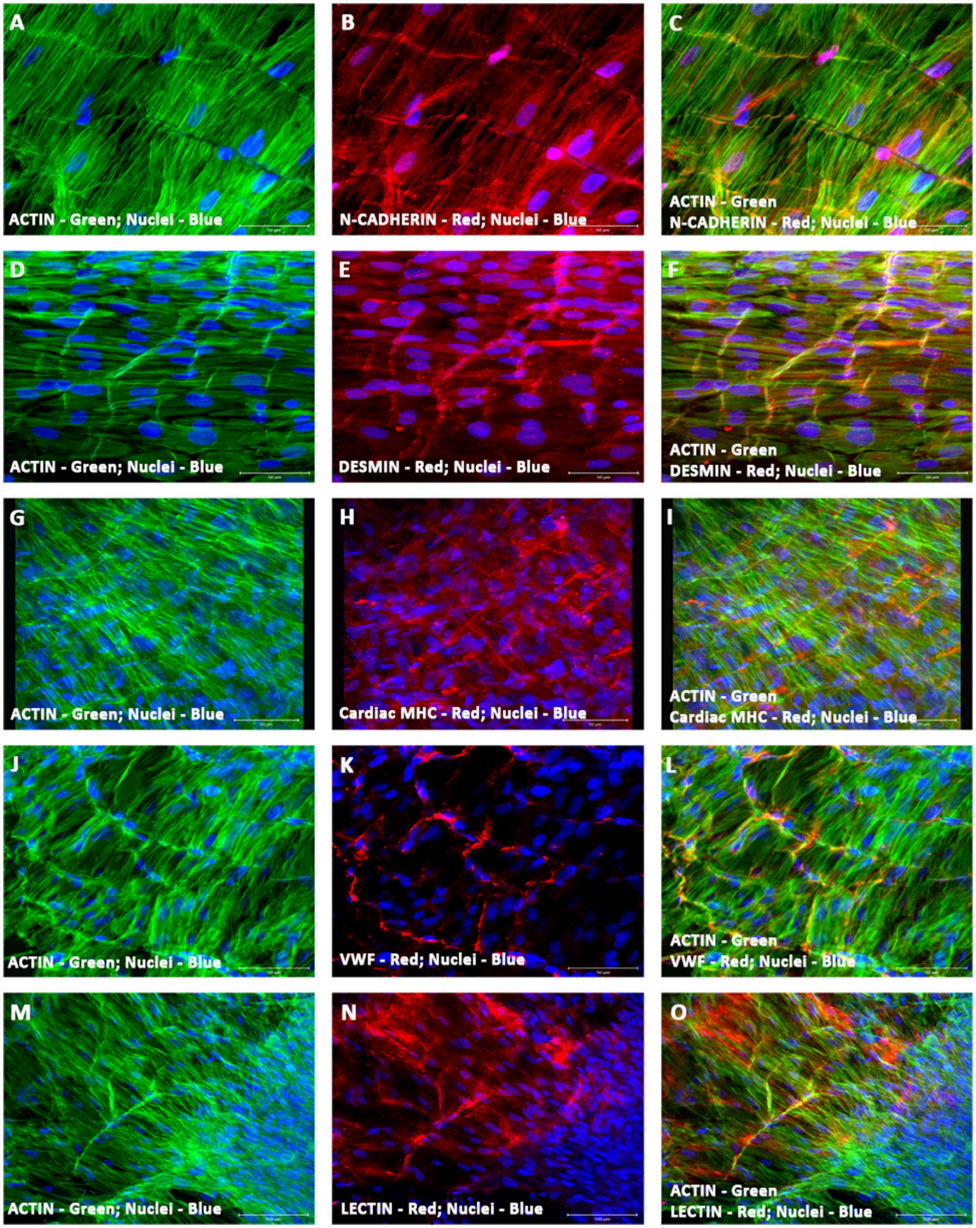
**Characterization of vascularized cardiac patch by confocal scanning laser microscopy**. Localization of key cardiac myocyte phenotypic markers of the vascularized cardiac patches (hCMVECs/hiPSC-ECMs/hMSCs co-culture) demonstrated the expression of structural and contractile proteins, N-Cadherin **(B,C)**, desmin **(E,F)**, cardiac MHC **(H,I)**, and actin **(A,C,D,F,G,I,J,L,M,O)**. Dual immunostaining of CCCs showed linear arrays of strap-like myocytes with cross-striations and uniform registry of sarcomeres. The sarcomeres were positive for cardiac MHC and desmin. N-cadherin revealed regions of intercalated disks, evoking a functional syncytium. Nuclei of these cells were large and oval in appearance, and were centrally situated. Furthermore, localization of key vascular phenotypic markers of the same vascularized cardiac patches depicted the expression of mature endothelium-associated proteins, VWF **(K,L)** and lectin **(N,O)**. Dual immunostaining of CCCs revealed myriad network of linear and/or branching microvascular structure interposed between the latticework of cardiac myocytes. Nuclei of these vascular cells were smaller and either elongated or fusiform in nature. Cells were also stained for nuclei (blue, DAPI), and fibrillary actin (green, Alexa 488 phalloidin). Merged images **(A–O)**. (**A–O**, scale bar 100 μm).

To identify and validate the phenotypic characteristics of co-differentiating hiPSC-ECMs/hMSCs in the environment of the microvascular structures, hiPSC-ECMs/hMSCs were co-cultured simultaneously on top of the prevascularized CCCs. Day 14 co-cultures harboring the three categories of cells, hCMVECs/hiPSC-ECMs/hMSCs were subjected to immunofluorescent staining and confocal microscopic analysis, by utilizing the same set of vasculogenic and cardiomyogenic differentiation markers as stated above. Comparable to what was discerned within hiPSC-ECMs CCC cultures, the differentiating cells were in turn positive for a set of cardiomyocyte lineage-specific markers, such as cardiac α/β-MHC, cardiac cTnI, desmin, Cx45, and Cx43 (Figures 12A–O). Illustrative staining pattern captured for the myocyte-specific proteins within the vascularized cardiac patches were shown in Figures 12A–O.

By cellular cohesion and alignment, the differentiating myocytes appeared to be very much elongated and were structurally arranged into interweaving bundles of myocytes, in this co-culture condition (Figures 12A–O). The structurally oriented and multilayered cells revealed cardiac specific sarcomeric organization, which was positive for both desmin and cardiac myosin heavy chain, MHC (Figures 12E,H). In addition, the intercalated discs were identified as prominently N-cadherin or desmin stained lines crossing fibers either transversely or in a staggered and zigzag manner (Figures 12B,C,E,F). Nuclei of these cells were large, oval in shape and were centrally situated in the cytoplasm (Figures 12A–O). In addition, the cells were positive for the gap junction proteins, connexin 45 and 43 (data not shown). The Gap junctions were a key component of the intercalated discs, and were believed to provide a low resistance pathway coupling adjacent cells, hence culminating into a functional syncytium.

Amidst these aligned cardiac myocytes, extensive network of linear and/or branching microvascular structures were localized. Comparable to what was noticed within hCMVECs/hMSCs co-cultures, the differentiating cells were positive for a battery of endothelial and smooth muscle cell markers, such as VWF, lectin, and α-SMA. Characteristic staining pattern captured endothelial-associated proteins within the vascularized cardiac patches were shown in Figures 12J–O.

### Pharmacological assay of vascularized cardiac patch

**Figure 13:**
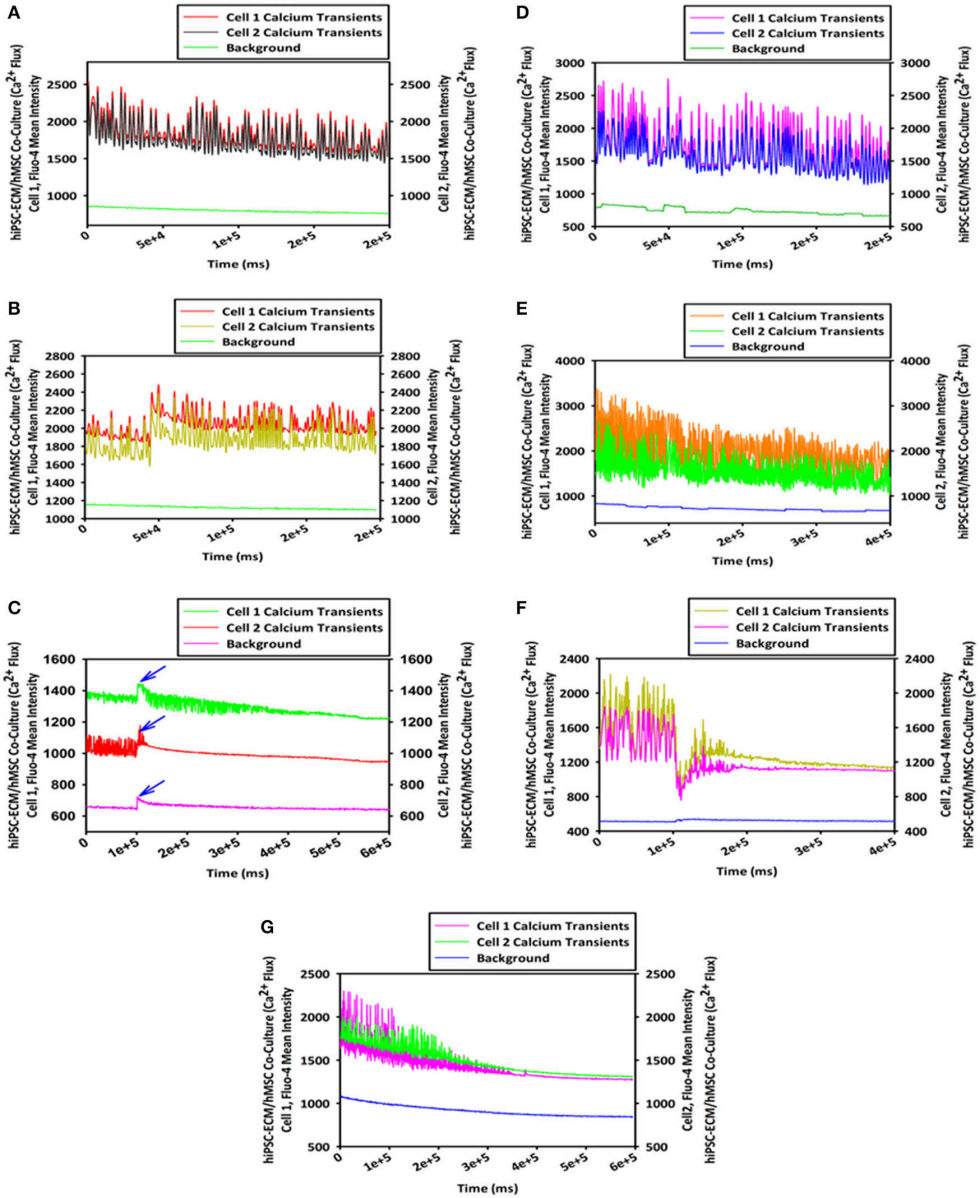
**Pharmacological assay of vascularized cardiac patch by live cell imaging with spinning disk confocal microscopy**. Analyses of the hCMVECs/hiPSC-ECMs/hMSCs co-culture derived vascularized cardiac patches in response to various cardioactive pharmacological agents (0.1 to 1 μM) displayed: Typical calcium oscillations of spontaneously and rhythmically beating cells, before the application of isoprenaline (a non-selective β adrenoceptor agonist), shown in **(A)**. Calcium oscillations in response to isoprenaline application revealed the typical elevation of chronotropic and ionotropic responses, shown in **(B)**. Calcium transient in response to isoprenaline followed by diltiazem, the initial application of isoprenaline to a beating cardiac patch displayed the typical elevated chronotropic and ionotropic responses (blue arrow). The subsequent application of diltiazem (a calcium channel blocker) led to the sustained negative chronotropic and ionotropic responses, shown in **(C)**. Typical calcium oscillations of spontaneously and rhythmically beating cells, before the application of clenbuterol (a sympathomimetic amine, β_2_ agonist), shown in **(D)**. Calcium flux in response to clenbuterol application demonstrated elevated ionotropic effect, shown in **(E)**. Calcium transient in response to clenbuterol followed by diltiazem, the initial application of clenbuterol to a spontaneously contracting cardiac patch exhibited enhanced ionotropic response. Whereas, an immediate application of diltiazem progressively abolished the beating of cardiac patch, shown in **(F)**. Calcium flux in response to application of diltiazem to a spontaneously and rhythmically beating cardiac patch resulted in negative chronotropic and inotropic responses, which ultimately led to the cessation of myocytes contractility, shown in **(G)**.

Analysis of the hCMVECs/hiPSC-ECMs/hMSCs derived vascularized cardiac patches in response to various cardioactive pharmacological agents displayed typical calcium flux, and hence the contractile response. Application of isoprenaline alone, a non-selective β adrenoceptor agonist, showed an increase in the frequency and amplitude of calcium flux, indicating an increase in the cardiac myocyte beating rate as well as its increased contractile force. Thus, the elevation of chronotropic and inotropic effects (Figures 13A,B). Whereas, application of diltiazem alone, a calcium channel blocker, led to an opposite effect. It progressively attenuated the frequency and amplitude of calcium flux, indicating a weakened cardiac myocyte beating rate and its decreased contractile force. Hence, the decreased chronotropic and inotropic effects (Figure 13G). Calcium transient in response to isoprenaline followed by diltiazem, the initial application of isoprenaline to beating cardiac patch displayed the typical elevated chronotropic and ionotropic responses (blue arrow). Then, followed by an immediate application of diltiazem showed sustained negative chronotropic and ionotropic responses (Figure 13C). Application of clenbuterol, a sympathomimetic amine (a β_2_ agonist) led to a raise in the frequency of calcium flux, thus, increase in the cardiac myocytes beating, thus an elevated inotropic effect (Figures 13D, E). Calcium transient in response to clenbuterol followed by diltiazem, the initial application of clenbuterol to beating cardiac patch displayed elevated inotropic effect. Then, followed by an application of diltiazem showed continued negative chronotropic and inotropic effects (Figure 13F).

## Discussion

Use of adult-tissue-derived stem cells in the stimulation of mammalian cardiac muscle regeneration is still in its early stages, and so far, it has been difficult to determine the efficacy of the procedures that have been employed (Jackson et al., 2001; Toma et al., 2002; Mangi et al., 2003; Balsam et al., 2004; Chen et al., 2004; Couzin and Vogel, 2004; Murry et al., 2004; Orlic, 2004; Laflamme and Murry, 2005). Collective evidence suggests that stem cells could play a vital role in cardiac regeneration, but this concept required further validation, since several critical issues remain unresolved until now. The outstanding question remains whether stem cells derived from the bone marrow or some other location within or outside the heart can populate a region of myocardial damage and transform into cardiac tissue-specific cells and also exhibit functional synchronization (Carlson, 2007). As a result, this necessitates the prompt development of appropriate *in vitro* 3-D model of cardiomyogenesis, and prompts us the development of a 3-D vascularized cardiac muscle construct for tissue engineering purposes, especially using the putative postnatal-/adult-tissue-derived stem cells, hiPSCs and hMSCs for personalized medicine.

Cumulative evidence suggests, using stem cells, it has been possible to stimulate mammalian cardiac muscle regeneration, and researchers have investigated the potential of various multi- and/or pluri-potential stem cells, such as MSCs, ESCs, iPSCs, and CPCs. Preliminary studies using small and larger animal models have indicated that bone marrow stromal cells/mesenchymal stem cells (BMSCs/MSCs), when transplanted into a cardiac lesion, can have certain degree of beneficial effects (Makino et al., 1999; Boyle et al., 2006). MSCs are multipotent, capable of differentiating into the prototypic cardiovascular cells, such as striated muscle cells, endothelial cells, and smooth muscle cells *in vitro* (Valarmathi et al., 2010, 2011), and contribute to myocardial regeneration *in vivo* when transplanted into the failing heart following myocardial infarction or non-infarction in mammalian model system, including human beings (Boyle et al., 2006; Makino et al., 1999). Nevertheless, for example, the central issue that remains to be addressed is the extent to which introduced stem cells and/or endogenous cardiac progenitor cells actually contribute directly to the formation neo-cardiomyocytes vs. their contribution to and/or stimulation of an enhanced local vascular response, which in turn may act as a supportive microenvironment for regenerative process (Orlic, 2004; Carlson, 2007), or whether the injected cells in fact accomplish myocardial repair by means of paracrine signaling, i.e., by secreting a cocktail of growth factors rather than essentially incorporating into the damaged myocardium, or by some other mechanism is actively under investigation (Carlson, 2007).

Angiogenesis and vascular invasion are a prerequisite to the process of tissue morphogenesis both in development and repair. Apart from their vital role in oxygen and nutrient delivery, it has recently been recognized that endothelial cells play an essential role in regulating and maintaining tissue-specific cells, and reported to influence both early cardiac development and in adult heart (Brutsaert, 2003; Cleaver and Melton, 2003; Hsieh et al., 2006). Previous studies indicate that microvascular endothelial cells promote cardiac myocyte survival and spatial reorganization (Brutsaert et al., 1998; Brutsaert, 2003; Narmoneva et al., 2004). In addition, *in vitro* heterotypic primary culture (co-culture) of microvascular endothelial cells and ventricular cardiac myocytes have demonstrated that reciprocal intercellular signaling regulates cardiac growth and function, and operates by means of autocrine and paracrine mechanisms (Nishida et al., 1993). Such intercellular signaling has also been shown to regulate cardiac myocyte contractility and apoptosis (Ramaciotti et al., 1992; Kuramochi et al., 2004). On the other hand, cardiac myocytes are presumed to influence endothelial cell survival and assembly. Taken together, these facts suggest that one of the successful strategies for myocardial regeneration may therefore depend on establishing functional myocyte-endothelium interactions.

Previously, our group has demonstrated the use of a novel 3-D aligned collagen-gel tubular scaffold in which rat embryonic cardiac myocytes could grow and maintain an *in vivo*-like phenotype, and recapitulates many aspects of developing myocytes (Evans et al., 2003). In our recent studies, we have characterized and demonstrated the osteogenic and vasculogenic differentiation potential of adult BMSCs when seeded onto a 3-D tubular scaffold and cultured in osteogenic growth medium (Valarmathi et al., 2008a,b). Similarly, we have shown that a pure population of adult BMSCs (CD90^+^) can be a potential cellular source for vascular tissue engineering using the same 3-D tubular scaffold. Our construct supported the development of in situ *de novo* microvascular structures under both vasculogenic and non-vasculogenic culture conditions (Valarmathi et al., 2009). We also explored the potential of the 3-D model system to recapitulate postnatal *de novo* vasculogenesis (Valarmathi et al., 2009).

Here we report first-time a reproducible and quintessential *in vitro* 3-D model of vascularized cardiac tissue composed of embryonic cardiac myocytes and cardiac-derived microvascular endothelial cells for exploring stem cell based cardiomyogenesis. This mosaic of cardiac tissue generated in this co-culture conditions recapitulated several aspects of *in vivo* neo-cardiomyogenesis, as well as allowed us to study the concurrent temporal and spatial regulation of cardiomyogenesis in the context of postnatal neo-vasculogenesis during stem cell cardiac regeneration. Thus, we have examined the temporal and spatial regulation of co-differentiating hiPSC-ECMs (myocytes) and hCMVECs (microvascular endothelial cells) in our CCC constructs in the presence of adult stem cell, hMSCs, and ultimately, their cell fate determined.

Our results have provided compelling evidence that presence of preexisting microvessels can able to accelerate and maintain the *in vivo* phenotype of ventricular cardiac myocytes. In addition, we have shown the progressive maturation and differentiation of these ventricular embryonic cardiac myocytes, from embryonic to neonatal type of phenotype. These myocytes expressed not only sarcomeric α/β-myosin heavy chain, a marker for mature myocytes but also revealed more uniform registry, and the presence of multiple intercalated discs. Finally, we have shown the presence of the gap junction protein, connexin 43 that is localized at the intercalated discs. Given the interplay of these two types of cells, viz., microvascular endothelial cells and embryonic cardiac myocytes in cardiac development, we have observed larger caliber vessels created in the CCC scaffold. Moreover, we have demonstrated unequivocally that hMSC-derived smooth muscle cells contributed to the development of tunica media of the larger caliber vessels, besides the presence of hMSC-derived neo-cardiomyocytes in this co-culture condition.

In general, any successful tissue engineering approach to regeneration depends not only on the right choice of substrate and/or engineered scaffold but most crucially on the right source of initial cells, which will ultimately dictate and fill the defects and/or repair the lesions. The cell carrier, CCC, is a novel, thin, and planar collagen scaffold. It is based on fibrillary bovine collagen type I and exhibits a low material thickness (40 μm) coupled with a high mechanical stability as measured by tensile tests (Schmidt et al., 2011). Both the mechanical properties and the *in vitro* biocompatibility of this CCC facilitate the engineering of thin transferable tissue constructs that offer new unlimited possibilities in the field of tissue engineering and regenerative medicine (Schmidt et al., 2011).

Considering the fact that hiPSC-ECMs and hMSCs may represent the cells of greatest potential for adult autologous and/or allogenic stem based cardiac regeneration, we have evaluated their integrative competence using developmental biology and tissue engineering principles. hiPSC-ECMs were seeded onto a 3-D biomimetic and biocompatible CCC scaffold together with hCMVECs in the presence or absence of hMSCs. The engineered vascularized cardiac tissue construct exhibited spontaneous beating and rhythmic contractions, and was observed using phase contrast time-lapse video microscopy as well as using spinning disk confocal microscopy during calcium spark imaging. Ultrastructural analysis and Immunohistochemical analysis using a battery of cardiac specific proteins confirmed the continuum of myogenic maturation and differentiation, i.e., progression from embryonic to neonatal phenotype. Furthermore, the spontaneously active cardiac patch briskly responded to various cardioselective drugs, both positive and negative chronotropic and ionotropic agents.

The advantages of the CCC model vs. other model systems are, the CCC's geometry and fiber topography allow for the production of vasculogenic structures in addition to various differentiated cells from a pure population of hMSCs not seen previously with any other scaffold. The CCC allows for flexibility and versatility when it comes to mechanical forces because the CCCs can be made to interface well with flow bioreactors and with the device tensile tester, and is more amenable to various mechanical stimuli. Thus, it is an ideal model system to examine further the effects of cell/cell and cell/mechanotransduction on the maturation of a vascularized cardiac construct, as it mimics developing embryonic vertebrate primitive heart tissue.

Since, in our previous studies, we have demonstrated that MSCs are not only capable of differentiating into endothelial and smooth muscle cells (Valarmathi et al., 2009) but also are capable of differentiating into myocytes under appropriate physicochemical and biological cues (Valarmathi et al., 2010, 2011). Therefore, further co-culture experiments with fluorescent protein labeled cells (for example, a combination of RFP-hiPSC-ECMs, GFP-hMSCs, and YFP-hCMVECs) are in progress, specifically to assess and delineate the exact fate and contribution of each of those three categories of cells in the cardiac patch. Efforts are also underway to innervate the cardiac patch with stem cell-derived autonomic neurons, i.e., the sympathetic and parasympathetic neuronal cells.

## Conclusions

Here we report the development of a reproducible *in vitro* 3-D model of cardiomyogenesis, as well as the generation of an archetypical 3-D vascularized cardiac patch for cardiovascular tissue engineering purposes. This study is innovative on several fronts, firstly, it uses co-culture of endothelial cells and cardiac myocytes to allow for cell-cell interactions that would exist in the heart during physiological (maintenance) and/or in pathological (reparative) regenerative processes. Second, a 3-D culture system to provide an *in vivo*-like cellular morphology and cell-matrix interactions. Next, the vascularization of the 3-D construct may allow for enhanced graft host integration on implantation, and eliminates the commonly encountered immunological barrier, since all sets of cells originate from the same species. Finally, a reproducible *in vitro* 3-D model of vascularized cardiac muscle construct for exploring adult stem cell based myocardial regeneration that can be monitored for all aspects of cardiac regeneration, e.g., neo-cardiomyocyte maturation and differentiation, excitation-contraction coupling, as well as cellular incorporation. Ultimately, the outcome from this study may translate to human medicine by providing a targeted approach to regulate the regenerative properties of myocardium and improved cardiac function post-MI.

## Author note

We confirm that MV, JF, JD, and RP meet the stipulated criteria for authorship of this original article based on the recommendation of the ICMJE.

## Author contributions

MV: Conception and design, financial support, provision of study material, collection and assembly of data, data analysis and interpretation, manuscript writing, final approval of manuscript. JF: Collection and assembly of data, data analysis and interpretation, manuscript editing, final approval of manuscript. JD: Collection and assembly of data, data analysis and interpretation, final approval of manuscript. RP: Administrative support, instrumentation resource facility, data interpretation, manuscript editing, final approval of manuscript.

## Funding

“This work was supported by an award from the American Heart Association.”—National Scientist Development Grant (11SDG5280022) for MV.

### Conflict of interest statement

The authors declare that the research was conducted in the absence of any commercial or financial relationships that could be construed as a potential conflict of interest.
